# Draft genome sequence and comparative genomic analysis of *Halomonas salifodinae* strain A2 isolated from the Zapotitlán Salinas Valley, Puebla, Mexico

**DOI:** 10.1007/s00792-025-01397-z

**Published:** 2025-07-03

**Authors:** Alberto León-Lemus, Martha Martínez-García, Nathalie Cabirol, Jorge E. Campos, Alejandro Monsalvo-Reyes

**Affiliations:** 1 Facultad de Estudios Superiores Iztacala, Laboratorio de Bioquímica Molecular de La Unidad de Biotecnología y Prototipos, Universidad Nacional Autónoma de México, Av. de los Barrios 1, 54090 Tlalnepantla, Estado de México México; 2 Posgrado en Ciencias Biológicas, Unidad de Posgrado, Universidad Nacional Autónoma de México, Edificio D, 1° Piso, Circuito de Posgrados, Ciudad Universitaria, Coyoacán, C.P. 04510 CDMX México; 3 Facultad de Ciencias, Departamento de Ecología y Recursos Naturales, Universidad Nacional Autónoma de México, Av. Investigación Científica, C.U., Coyoacán, 04510 CDMX México

**Keywords:** Biotechnology, *Bisbaumannia*, Compatible solutes, *Halomonas*, Osmoadaptation, Reclassification

## Abstract

In this study, we report the draft genome sequence of strain A2. The genome size was 3.8 Mbp, the GC content was 67.4%, and it was predicted to contain 3520 protein-coding genes, 62 tRNA genes, 8 rRNA genes, and 4 snRNA genes. Phylogenetic analysis of the 16S rRNA gene in different databases suggests that strain A2 belongs to *Halomonas salifodinae*. Also, Multilocus Sequence Typing analysis confirms that A2 is closely related to *H. salifodinae*. Phylogenomic of the core genes and comparative genomic analysis using the Average Nucleotide Identity, digital DNA-DNA Hybridization, Average Amino acid Identity, the Percentage of Conserved Proteins values indicators, and the Genome Taxonomy Database indicates that strain A2 is identified as *H. salifodinae* and suggest that this species has a closer phylogenetic relationship with the genus *Bisbaumannia* than with *Halomonas*. The pangenomic analysis of A2 against 100 reference genomes of described *Halomonas* and another related genus shows 136,122 genes that comprise the pangenome with 317 core genes, 3457 shell genes, 132,332 accessory genome genes, and 691 unique genes. A2 has 261 signature genes that it shares only with *Bisbaumannia* and *Halomonas salifodinae.* For strain A2 we found 29 genes for secretion systems, 23 genes for Na^+^ and K^+^ ion transport, 6 Biosynthetic Gene Clusters groups, a total of 12 genomic islands, an 8.2 kb gene prophage region, 15 regions associated with CRISPR and one CAS-TypeIF *cas* gene cluster region, 12 genes of biotechnological importance, 38 unique genes essential for adaptability and biotechnological relevance, as well as, 35 genes for the synthesis of compatible solutes. Comparative genomics analysis shows that strain A2 has multiple unique genetic features that could be useful for biotechnological applications. The result of this study places the species *Halomonas salifodinae* in a very close relationship with the genus *Bisbaumannia* than with *Halomonas*, so its reclassification to the genus *Bisbaumannia* is proposed for future validation.

**Supplementary Information:**

The online version contains supplementary material available at 10.1007/s00792-025-01397-z.

## Introduction

Halotolerant or halophilic microorganisms, capable of living in saline environments, offer many potential applications in various fields (Margesin and Schinner 2001). Extreme environments like saline lakes, coastal lagoons, and artificial salt mines contain highly concentrated salt solutions and support only life forms adapted to these adverse conditions. However, these environments typically harbour dense microbial populations consisting of species from all three domains of life (Oren et al. 2008). Moderately halophilic microorganisms can grow optimally in media containing 3–15% NaCl (Kushner et al. 1988), although most can grow in an extensive range of salinities (Ventosa et al. 1998).

To withstand these conditions, bacteria accumulate organic compounds called compatible solutes, which are highly soluble in water and maintain an osmotic balance with the surrounding medium (Brown 1976; Litzner et al. 2006; Roberts 2005). Compatible solutes benefit bacterial cells as osmoregulatory solutes and protein protectants by mitigating the detrimental effects of freezing, desiccation, and high temperatures (Borges et al. 2002; Clegg et al. 1982).

It has been proposed that compatible solutes can counteract osmotic inhibition by increasing the overall water content and, consequently, the cytoplasmic volume of cells. (Clegg et al. 1982; Galinski et al. 1997; Kolp et al. 2006). The structure-forming and breakage properties of compatible solutes indirectly influence the hydration “shells” and, therefore, the activities of the proteins involved (Arakawa and Timasheff 1985; Bolen et al. 2001; Di Gioacchino et al. 2019; Kurz 2008; Liu and Bolen 1995; Timasheff 2002). The predominant compatible solutes in halophilic bacteria are amino acid derivatives, such as ectoine. (Oren et al. 2008; Roberts 2005). Many bacteria synthesise the aspartate derivative ectoine (1,4,5,6-tetrahydro-2-methyl-4-pyrimidinecarboxylic acid) as one of their primary compatible solutes (Roberts 2005). Ectoine is synthesised from aspartate-semialdehyde and comprises three enzymatic steps; the genes involved in ectoine synthesis are located continuously in the *ect*ABC operon (Ono et al. 1999). There is a wide range of technological and industrial applications for ectoine and other compatible solutes, starting with cosmetics such as sunscreens and anti-ageing products (Bujak et al. 2020; Heinrich et al. 2007; Ma et al. 2022). Treatments of inflammatory skin conditions such as topical dermatitis, dry eye, dry nose, and rhinosinusitis have been developed and successfully tested in clinical trials (Eichel et al. 2013; Dao et al. 2019). They are also protecting DNA against ionising radiation during radiotherapies and chemotherapies in cancer patients (Dao et al. 2018; Refaat et al. 2018; Rieckmann et al. 2019; Schröter et al. 2017). It has been tested as a protein stabiliser in experimental therapies against neurodegenerative diseases such as Alzheimer’s, Parkinson’s, and Huntington’s (Arora and Park 2004; Ashraf et al. 2014; Bazazzadegan et al. 2017; Furusho and Shoi 2005; Kanapathipillai et al. 2008; Yang et al. 1999). In addition, a new application for producing ectoine using biogas promotes a green and circular economy (Cantera et al. 2020; Pérez et al. 2022).

Complete or partial sequencing of bacterial genomes benefits functional studies of compatible solutes such as ectoine (Imhoff et al. 2020; Zhang et al. 2022). The bacterial genome sequences provide valuable biological information, including unique genes and their functions, the presence and absence of specific genes, and other hidden characteristics within the genomes. (Gao et al. 2015). Among halophilic microorganisms, some prominent characteristics found in their genome include protein secretion systems, ion transport proteins, secondary metabolites, genomic islands, prophage sequences, CRISPR/Cas system sequences, and a diversity of genes with biotechnological and environmental significance.

The genetic modification of protein secretion systems in model microorganisms such as *Bacillus subtilis* and *Escherichia coli* has proven successful in various industrial applications. It has revealed valuable information about the structure and function of the components of secretory system machinery (Lin et al. 2021). 

Ion transport proteins are essential to survival in saline environments because high intracellular potassium and low sodium concentrations must be maintained for effective cellular enzymatic activity (Kraegeloh and Kunte 2002). These systems work together with compatible solutes to regulate intracellular osmotic pressure. Chen et al. (2017) propose that organisms with these underlying salinity tolerance mechanisms are especially attractive from an industrial perspective.

Microbial secondary metabolites are widely used in medicine, agriculture, and industry and are known to mediate various interactions between microbes and their environment. The understanding of the genes that encode them is revolutionising their study, enabling the discovery of new molecules within genomes (Cimermancic et al. 2014).

Horizontal gene transfer is an essential mechanism for microbial genome evolution, allowing rapid adaptation and survival in specific niches. Genomic islands are commonly defined as groups of bacterial or archaeal genes that have likely been transferred horizontally. They are interested in medicine, environmental science, and industry (Bertelli et al. 2019).

Due to their ability to exchange genes, prophages play a crucial role in bacterial adaptation to compete with other bacteria and adjust metabolism depending on the environmental conditions in which they are found to survive and grow (Bondy-Denomy and Davidson 2014).

The biotechnological applications of bacterial CRISPR/Cas (Clustered regularly interspaced short palindromic repeat sequences) systems are progressing relatively quietly but steadily. There is a wider variety of CRISPR/Cas-based applications for bacteria, and all have great potential to improve bacteria-based industries (Choi and Lee 2016).

Natural polyesters such as polyhydroxyalkanoates (PHA) are produced and accumulated within bacterial cells. Their physicochemical and thermal properties depend on the characteristics of the producing organism and the culture conditions. It is important to evaluate the diversity of PHA-producing bacteria, their genetic characteristics, and the benefits and limitations they offer (Vicente et al. 2023).

The *Halomonas* genus (Vreeland et al. 1980; emendation: Dobson and Franzmann 1996) includes Gram-stain-negative straight or curved rods that can be motile or non-motile. Catalase-positive and oxidase-variable. Strictly aerobic or facultatively anaerobic. Some species are haloalkaliphilic or psychrotolerant. Optimal growth at 0–15% NaCl, pH 6.0–10.0, and 20–40 °C. Halophilic or halotolerant. Some species have been reported to grow under anaerobic conditions in the absence of nitrate if supplied with glucose (but not other carbohydrates or amino acids). Colonies are cream, cream-yellow, yellow, white, brown, or orange pigmented. Endospores are not formed. Chemoorganotrophic. Carbohydrates, amino acids, polyols, and hydrocarbons can serve as sole carbon sources in mineral media. Ammonium sulfate can serve as a sole nitrogen source. DNA G + C content 51.4–74.3% (Ventosa et al. 2021; Vreeland and Martin 1980). It is one of the most representative groups of bacteria within moderate halophilic organisms (Canovas et al. 1996); In accordance with the LPSN it belongs to the phylum *Pseudomonadota* (Oren and Garrity 2021), class *Gammaproteobacteria* (Garrity et al. 2005b), order *Oceanospirillales* (Garrity et al. 2005a), family *Halomonadaceae* (de la Haba et al. 2023; Franzmann et al. 1988).

The Tehuacán–Cuicatlán Valley, located in central Mexico, is considered a center of megadiversity and endemism worldwide by the International Union for the Conservation of Nature (IUCN) (Dávila et al. 2002). The Zapotitlán Salinas Valley is located within this territory, which has a tremendous biotic diversity of dry areas with multiple biological forms and varied expressions of adaptation strategies. The climate is dry semi-warm, with summer rains, and the average temperature fluctuates between 17.6 and 23.7 °C. From the edaphic point of view, the soils are shallow and stony in most of the area, with different levels of alkalinity and salinity resulting from the influence of the different geological substrates derived from the Lower and Middle Cretaceous (Dávila et al. 2002). The hydrogeochemistry of this site shows a high concentration of NaCl and other salts (Villa et al. 2014). The area is characterised by xeric scrub vegetation, dominated by columnar cacti (Rzedowski and Calderón, 1994).

The genome of a halophilic microorganism can be analysed in detail to reveal its potential biotechnological applications. Furthermore, genome sequencing can provide phylogenetic information that helps to improve understanding of the taxonomic position of the species. The genus *Halomonas* has not been phylogenetically consistent; for a long time, new species have been continuously described within the family *Halomonadaceae* that could not be classified in another genus outside *Halomonas*. However, de la Haba et al. (2023) used multiple OGRIs (Overall Genome Relatedness Indexes) and pangenome analysis reorganised the taxogenomics of *Halomonadeceae*, but some species whose genomes were not available at the time of the study could not be reclassified, such as the case of *Halomonas salifodinae*. In this study, we sequenced and analysed the genome of a moderately halophilic bacterium, strain A2, which was isolated from the water of the Salinas de Zapotitlán Valley in Puebla, Mexico. *Halomonas* species have not previously been isolated in this geographic area, and this is the first time such an isolation has been achieved. All phylogenomic and phylogenetic analyses conducted indicate that strain A2 is classified as belonging to the species *Halomonas salifodinae*. Furthermore, these analyses suggest that the species has a much closer relationship with species of the genus *Bisbaumannia*. The genome contains several genes that are related to adapting to saline environments. It also includes genes for secretory proteins, ion transport, secondary metabolites, genomic islands, CRISPR-Cas regions, prophage sequence regions, genes of biotechnological importance, and biosynthetic genes for compatible solutes. Additionally, a pangenome analysis, detection of unique genes and signature genes were performed.

## Materials and method

### Bacterial strain and culture

Strain A2 was isolated from a water sample from the “Las Chiquitas” salt mine (18°20′49.0ʺ N—97°26′59.0ʺ W) in the Zapotitlán Salinas Valley Puebla, Mexico. The sampling site conditions were pH 9.8, atmospheric temperature 28.2 °C, and water temperature 22.5 °C. Strain A2 was isolated by taking 1 ml of water from the sample and diluting it 1:10 in 5% saline solution for inoculation. The culture medium was BD Bioxon^®^ solid nutrient medium (Becton Dickinson, USA) containing 5.0 g pancreatic digest of gelatine, 3.0 g beef extract, 15.0 g agar, and we added 10% NaCl (1.7 mol/l). The pH of the medium was 7.8. To determine the optimal growth of the strain, growth assays were conducted at various salinity levels (0–20% NaCl, 0–3.5 mol/l) at 35 °C under aerobic conditions for 36 h.

### Bacterial cell morphology

Strain A2 was cultured in a nutrient medium with 1.7 mol/l NaCl at 35 °C for 36 h. Cell morphology was determined by Gram staining using light microscopy at × 400 and × 1000 magnification. Cells were prepared for transmission electron microscopy (TEM) by performing conventional fixation with glutaraldehyde and aqueous osmium tetroxide and dehydrating the sample with ethanol. Electron micrographs were prepared using a JEOL^®^ TEM model JEM-1010 with an acceleration voltage of 80 kV.

### DNA extraction and genome sequencing

The strain was grown in a nutrient medium with 1.7 mol/l NaCl at 35 °C for 36 h. It was harvested during the logarithmic growth phase, stored at –70 °C in 2 ml tubes, and then sent on dry ice for processing. Novogene Co. America (CA, USA) performed DNA extraction and genome sequencing on the Illumina Novaseq 6000 platform. The obtained fragments were end-repaired, A-tailed and further ligated with Illumina adapter; the raw data were cleaned until a Clean Data Q20(%) 97.31 and Q30(%) 92.6 were obtained. Genome assembly was performed using SOAPdenovo software version 2.0 (Luo et al. 2012), SPAdes version 3.15.1 (Prjibelski et al. 2020), and Abyss software version 2.0. (Jackman et al. 2017). The assembly results from the three software were integrated with the CISA software (Lin and Liao 2013), and the resulting assembly with the least number of scaffolds was selected. Genome quality was measured on the MiGA server (Rodriguez-R et al. 2020).

### Gene prediction and annotation

The prediction of genome components included, coding genes, repetitive sequences, non-coding RNA, genomic islands, prophages regions, CRISPR, and secondary metabolism genes, was performed as follows: GeneMarkS program server version 4.28 (Besemer et al. 2001) was employed to retrieve related coding genes, interspersed repetitive sequences were predicted using RepeatMasker server version 4.1.3 (Saha et al. 2008), tandem repeats were analysed by the server tandem repeat finder (TRF) (Benson 1999), tRNA genes were predicted by tRNAscan-SE version 2.0 (Chan and Lowe 2019; Lowe and Chan 2016), rRNA were analysed by rRNAmmer-1.2 (Lagesen et al. 2007), BLAST predicted small nuclear RNAs (snRNAs) against the Rfam database (Gardner et al. 2009), the Islandviewer 4 (Hsiao et al. 2003) was used to predict Genomic Islands and PHASTER (Arndt et al. 2016) was used for prophage prediction. CRISPRFinder (Grissa et al. 2007) was used for CRISPR identification, and we analysed secondary metabolism gene clusters using antiSMASH 7.0 (Blin et al. 2023).

For gene annotation, we used Diamond version 0.8.35 (Buchfink et al. 2021), hmmer3 version 3.3.2 (Eddy 2011) and Blast2go (version 2.5). We used six databases to predict gene functions in Gene Ontology (GO), Kyoto Encyclopaedia of Genes and Genomes (KEGG), Clusters of Orthologous Groups (COG), Nonredundant Protein Database (NR), Protein Family Database (Pfam) and Swiss-Prot. A whole-genome blast search (E value less than 1e^−5^, per cent minimum alignment length greater than 40%) was performed on the above six databases. We manually searched for genes related to survival in saline environments, biosynthesis of compatible solutes, and other genes with biotechnological importance using the annotations provided by the KEGG and NR databases. We confirmed our findings by conducting local alignments with the BLAST^®^ tool at the National Center for Biotechnology Information (NCBI).

### Phylogenetic and phylogenomic analysis

#### 16S rRNA gene analysis

For phylogenetic analysis, the sequence of the 16S rRNA gene of A2 was compared in the databases of the NCBI, EzBioCloud, and Type Strain Genome Server (TYGS) (Meier-Kolthoff et al. 2022). The sequences with high similarities were obtained from the NCBI GenBank, and *Pseudomonas aeruginosa* strain DSM 50071^ T ^(NR_026078.1) was used as the outgroup. The MEGA11 software (Tamura et al. 2021) and Multiple Sequence Alignment by CLUSTALW provided by the GenomeNet server were used to align, compare, and construct the phylogenetic tree using the Neighbour Joining (NJ) method with Bootstrap of 1000.

#### Multilocus sequence typing analysis

To corroborate the taxonomic classification of A2, another phylogenetic tree of Multilocus Sequence Typing (MLST) genes (Florida-Yapur et al. 2021) was constructed using the genome of A2 in the autoMLST server (Alanjary et al. 2019), using the Denovo-workflow with 100 MLST genes suggested by default against closely related species; *Pseudomonas aeruginosa* strain DSM 50071^ T^ (NR_026078.1) was selected as an outgroup and *Bifidobacterium pseudolongum* strain DSM 20099^ T^ (GCF_000771225) was suggested by the server as default outgroup. The phylogenetic tree of the MSLT genes was performed by IQ-TREE Ultrafast Bootstrap analysis (1000 replicates) run ModelFinder to find the optimal model for tree building, and it was visualised and improved in the MEGA 11 software.

#### Overall genome relatedness indexes analysis

The taxonomic analysis of the complete A2 genome was performed on the TYGS server (Meier-Kolthoff et al. 2022). GTDB Anchor tool of the MiGA server was used to identify the closest relative in the Genome Taxonomy Database (GTDB) (Rodriguez-R et al. 2020). The Average Nucleotide Identity (ANI) values were calculated using FastANI version 1.34 (https://github.com/ParBLiSS/FastANI) with default settings. Average Amino Acid Identity (AAI) was calculated using the Enveomics package (Rodriguez-R and Konstantinidis 2016) with Diamond as protein mapper. We created a heat map on the Heatmapper server (Babicki et al. 2016) using the ANI and AAI value matrix results. The digital DNA-DNA Hybridization (dDDH) values were calculated in the Genome-to-Genome distance calculator (GGDC) server 3.0 (Meier-Kolthoff et al. 2022). The calculation of the Percentage of Conserved Proteins (POCP) values was obtained using the BLASTP aligner with the “POCP.sh” script (Moose 2017), written based on Qin et al. 2014. We compared the protein content of A2 and nine closely related species using BLASTP in the Proteome Comparison Tool on the Bacterial and Viral Bioinformatics Resource Center (BV-BRC) server (Olson et al. 2023).

#### Pangenome analysis

Pangenome construction was performed using rapid large-scale prokaryote pangenome analysis in Roary software version 3.11.2 (Page et al. 2015) using the GFF3 files provided by the Prokka software (Seemann 2014). For the core genome analysis, we used IQ-TREE server version 2.3.4 (Trifinopoulos et al. 2016) to construct the Maximum Likelihood phylogenetic tree using the General Time-Reversible Model (GTR) (Lanave et al. 1984) using an Ultrafast Bootstrap of 1000. Based on de la Haba et al. 2023, we used *Terasakiispira papahanaumokuakeensis* strain PH27A^T ^as outgroup. A search was carried out in the absence-presece gene matrix for signature genes specific to the genus, as suggested by de la Haba et al. 2023, to have more certainty in the phylogeny of A2. In addition, we searched for and identified unique genes specific to the strain that had biotechnological importance. 

## Results and discussion

### Strain A2 growth conditions

Strain A2 was grown in the nutrient medium at different concentrations of NaCl (0–20%, 0–3.5 mol/l) at 35 °C, showing optimal growth at 1.7 mol/l NaCl (10%) concentration. The strain showed observable growth after 36 h in a concentration range of 0.5–2.6 mol/l (3–15%) NaCl. In the 0–0.5 mol/l concentration and beyond 2.6 mol/l concentration, the growth was not detectable in the same time range.

### Cellular and colonial morphology

**Fig. 1:**
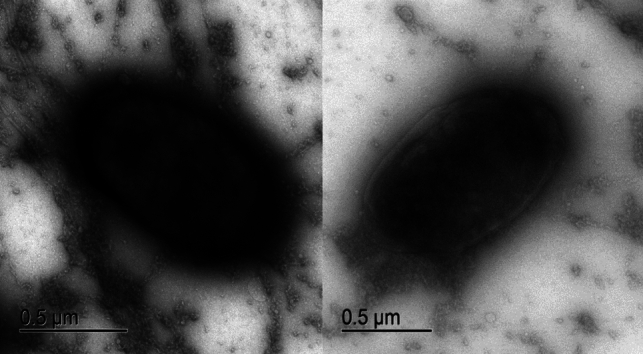
Transmission electron microscopy images of A2. In the micrograph, the morphology of coccobacillus of A2 is observed and shows a size of 1 µm approximately

The colonies of strain A2 had an indefinite morphology with an irregular light brown growth pattern and a slightly viscous consistency that expanded in all directions. For the cellular morphology of A2, we observed small cells around 1 µm in length (Fig. 1) and a coccobacillus morphology that reacted negatively to Gram staining (Fig. S1).

### Genome overview

The genome sequence of A2 was 3.8 Mbp in 33 contigs with a GC content of 67.5%; the genome is not closed. This is within the range reported to closely related species (Table S1). The genome of A2 contains 3,610 genes, of which 3,520 are protein-coding genes, 74 RNA genes and 13 pseudogenes. In the genome of A2, there are a total of 74 RNA-coding genes, of which 62 are tRNA genes, eight are rRNA genes, including one 16S rRNA, one 23S rRNA, and six for 5S rRNA; the remaining four correspond to snRNA. Furthermore, 303 tandem repeat sequences, 211 minisatellites, and five microsatellites were found. Six databases were used to annotate the functions of the coding sequences (CDSs). The genome sequence quality measured in MiGA shows indices of completeness 100% (very high), contamination 2.8% (very low) and Quality 86% (excellent). The total number of genes annotated by each of the GO, KEGG, COG, NR, Pfam, and Swiss-Prot databases were 2,557, 3,380, 2,967, 3,405, 2,557, and 1,848 respectively (Table 1).

**Table 1:** Project information and genome statistics

Attributes	Value
Sequencing platform	Illumina
Genome size (bp)	3,855,926
GC content %	67.4
Contig Max Length (bp)	483,539
Contig Min Length (bp)	648
Contigs	33
N50 Length(bp)	336,455
N90 Length (bp)	84,770
Scaffolds	0
Number of coding genes	3,520
RNA genes	74
tRNA	62
rRNA genes	8
5S rRNA	6
16S rRNA	1
23S rRNA	1
snRNA	4
Pseudogenes	13
GO annotation	2,557
KEGG annotation	3,380
COG annotation	2,967
NR annotation	3,405
Pfam annotation	2557
Swiss-Prot annotation	1848

### Genome annotation

For A2, the GO annotations (Fig. S2 **a**) are in three main categories: “molecular function” (3167), “cellular component” (2303), and “biological process” (5552). In the “molecular function” category, the five subcategories with the most annotations were “catalytic activity” (1380) and “binding” (1173). “Cell part” and “cell” were the subcategories that presented the most annotations, with 949 each in the “cellular component” category. In the “biological process” category were the “metabolic process” (1467), “cellular process” (1401), “localisation” (569), and “establishment of localisation” (552).

Based on sequence homologies, the genome was mapped to 24 categories in COG (Fig. S2 **b**). Regarding the above, “amino acid transport and metabolism” (E), “general functions” prediction (R), “energy production and conversion” (C), “translation, ribosomal structure, and biogenesis” (J), “transcription” (K) and “inorganic ion transport metabolism” (P) were the most abundant categories with 9.9%, 8.9%, 7.0%, 6.9%, 6.9%, and 6.0% respectively. These functions are essential for halophilic bacteria survival in hypersaline environments (Zhang et al. 2022).

The KEGG annotations (Fig. S2 **c**) have six categories with many annotated genes. For the “metabolism” category, we have amino acid and carbohydrate metabolism, which have the highest proportion of annotated genes, with 250 and 191, respectively. Regarding the annotation of amino acid metabolism, we can mention metabolic routes such as alanine, aspartate, and glutamate metabolism (ko00250), arginine and proline metabolism (ko00330), lysine biosynthesis (ko00300) and cysteine and methionine metabolism (ko00270), all of them closely related to the metabolic pathway of degradation and biosynthesis of ectoine and other compatible solutes, which is immersed in the glycine, serine and threonine metabolism pathway (ko00260). Pyruvate metabolism (ko00620) has the highest amount of carbohydrate metabolism annotations, glyoxylate and dicarboxylate metabolism (ko00630) have a second place and are followed by propanoate metabolism (ko00640) and butanoate metabolism (ko00650) respectively.

### Phylogenetic and phylogenomic analysis

#### 16S rRNA gene and MLST analysis

**Fig. 2:**
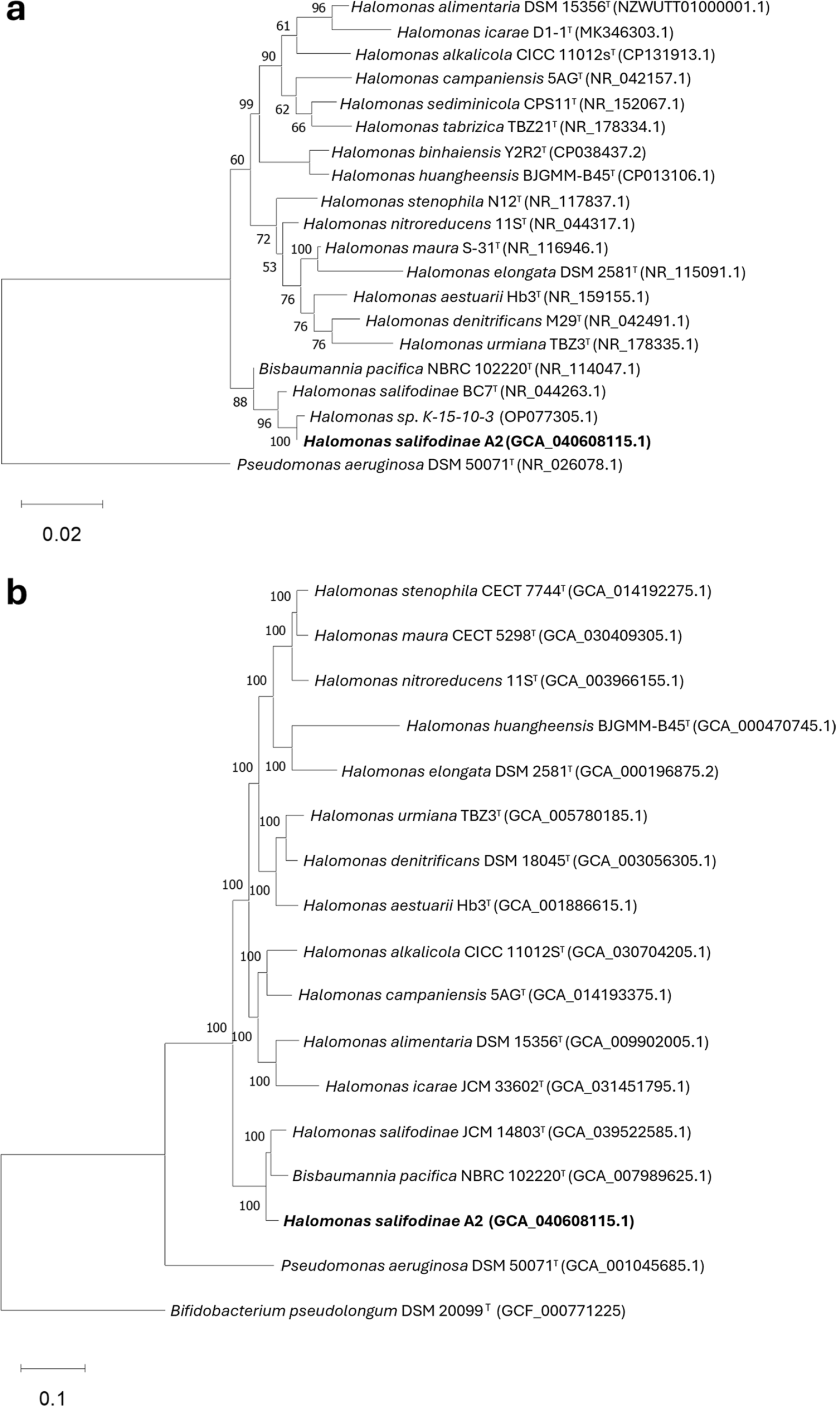
**a** Phylogenetic tree based on 16S rRNA sequences showing the phylogenetic position of A2. The phylogenetic tree was constructed using the NJ method bootstrap values were calculated from 1000 samples; **b** phylogenetic tree based on MLST gene sequences showing the phylogenetic position of A2, performed by IQ-TREE Ultrafast Bootstrap analysis (1000 replicates) run ModelFinder

**Fig. 3:**
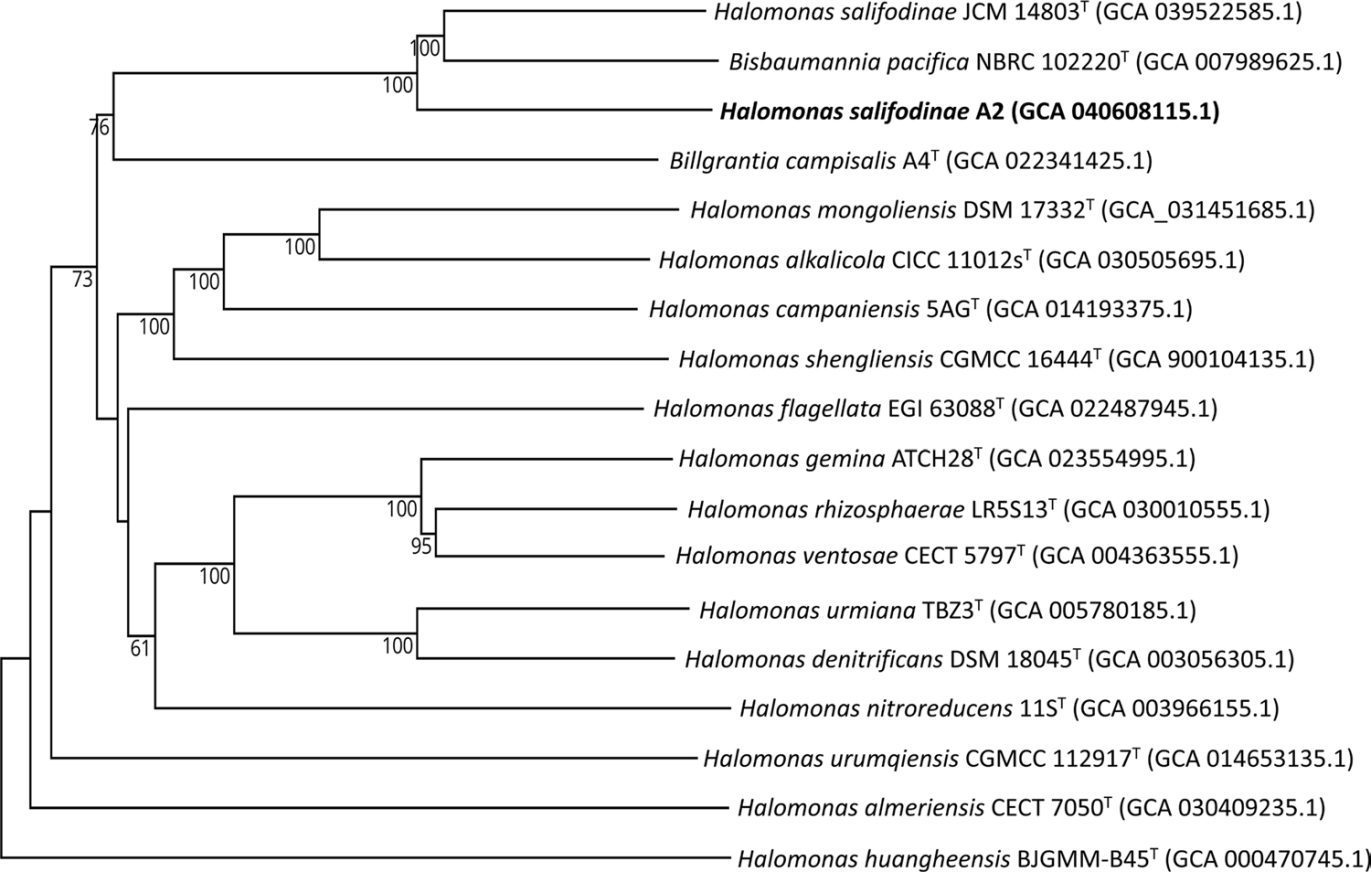
The genome-scale GBDP (Genome BLAST Distance Phylogeny) tree is based on comparing A2 genome and genomes from the TYGS database. Tree inferred with FastME 2.1.6.1 from GBDP distances calculated from genome sequences. Branch lengths are scaled in terms of the GBDP d5 distance formula. The numbers above branches are GBDP pseudo-bootstrap support values > 60% from 100 replications, with average branch support of 88.7%

The results of 16S rRNA gene sequence analysis showed that A2 belongs to the genus *Halomonas* with 99.9% similarity in the NCBI database with *Halomonas* sp. strain K-15–10-3 (OP077305.1), 99.2% with *Halomonas salifodinae* strain BC7^ T^ (EF527873.1) and 98.98% with *Bisbaumannia pacifica* strain NBRC 102220^ T^. All sequences were partial but almost complete above 1400 bp. However, in the new classification by de la Haba et al. (2023), *H. pacifica* becomes *Bisbaumannia pacifica*. For type species in the EzBiocloud database, there is 99.3% similarity with *H. salifodinae* BC7 ^T^, 98.9% with *B. pacifica* NBRC 102220^ T^, and 97.6% with *Halomonas sediminicola* CPS11^T^ (NR_152067.1). The phylogenetic tree (Fig. 2a) constructed with the results from the NCBI database shows the close relationship of A2 with *H. salifodinae* BC7 ^T ^and *B. pacifica* NBRC 102220^ T^, forming a branch apart from the other species. The phylogenetic tree constructed with the MLST genes (Fig. 2b) also results in a high similarity between A2 and *H. salifodinae* JCM 14803^ T ^(GCA_039522585.1), and both show a close relationship with *B. pacifica* NBRC 102220^ T^. In the phylogenetic analysis of the 16S rRNA gene sequences (Fig. S3) and the genome-to-genome comparison (Fig. 3) that was carried out in TYGS, the results are similar and corroborate the phylogenetic closeness of A2 with *H. salifodinae* JCM 14803^ T ^and *B. pacifica* NBRC 102220^ T ^that found in the same branch.

#### OGRIs analysis

**Fig. 4:**
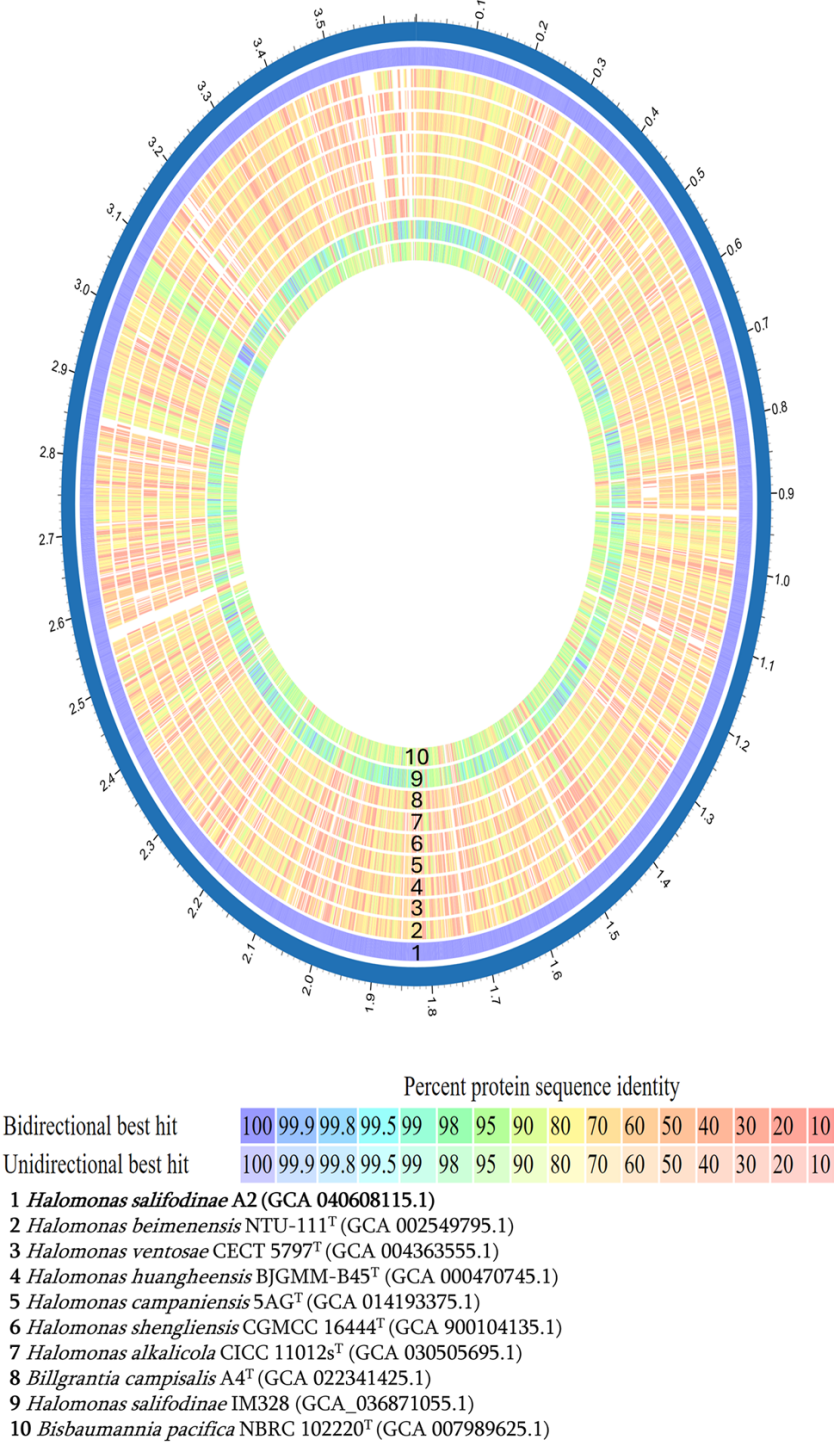
Protein contents comparison of A2 using BLASTP against closely related species

**Fig. 5:**
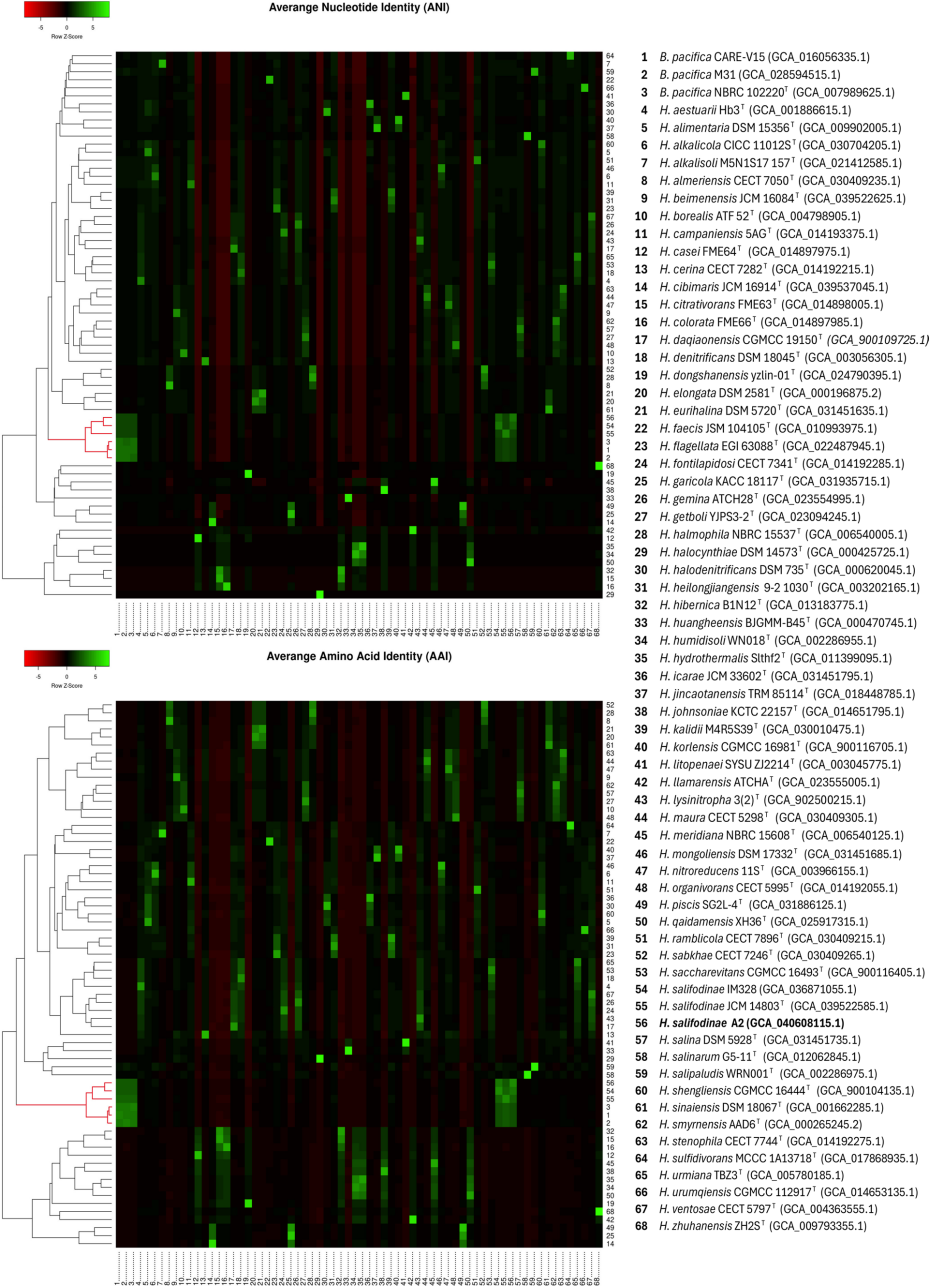
Heat maps illustrate the bidirectional matrix obtained from the ANI and AAI values that include A2 and the type species of *Halomonas* and *Bisbaumannia.* The branch formed by the strains of *Halomonas salifodinae* and *Bisbaumannia pacifica* was indicated in red

The taxonomic classification of the GTDB Anchor of the MiGA server identified strain A2 in the same group with *Bisbaumannia* sp015645095 ^T ^(GCF_015645095.1) with a 98.48 AAI identity in the GTDB database. The protein contents comparison using BLASTP of A2 shows considerably higher similarity to *H. salifodinae* IM328, followed by that of *B. pacifica* NBRC 102220^ T^, compared to closely related *Halomonas* species (Fig. 4). Based on the above data the genome of A2 was compared with genomes of *Halomonas* species using ANI, AAI, dDDH and POCP analysis (Table 2, Fig. 5). The ANI, AAI, dDDH and POCP values between A2 to *H. salifodinae* IM328 (GCA_036871055.1) were 96.5%, 96.7%, 66.8% and 91.89%, and between A2 to *Halomonas salifodinae* JCM 14803^ T ^were 91.8%, 91.5%, 42.2% and 82.03% respectively. At the same time, *B. pacifica* NBRC 102220^ T^ were 91.2%, 90.9%, 39.8% and 85.07%, respectively; the ANI, AAI, dDDH and POCP values for the other species did not exceed 84.2%, 76.5%, 24.8% and 71.3%, respectively. According to species delimitation, cut-off values of 95–96% for ANI and 70% for dDDH (Chun et al. 2018; Meier-Kolthof et al. 2022; Riesco and Trujillo 2024) and supported by the previous information, *Halomonas* sp. A2 is identified as *Bisbaumannia salifodinae* strain A2. The POCP and AAI values show that *Halomonas salifodinae* has a closer relationship with the genus *Bisbaumannia* than with *Halomonas* (Qin et al. 2014; Riesco and Trujillo 2024). Previous studies, such as the one carried out by de la Haba et al. 2023, show the importance of comparative genomics and the use of multiple OGRIs in the reclassification of multiple species of the genus *Halomonas* into seven new genera. However, the species *Halomonas salifodinae* was not included in the study because there was no sequenced genome. Our results place *Halomonas salifodinae* in a very close relationship with the genus *Bisbaumannia* than with the genus *Halomonas*, this suggests a reclassification of the species *Halomonas salifodinae* to the genus *Bisbaumannia*.

**Table 2:** The ANI, AAI, dDDH and POCP analysis between A2 and its closely related species

Hit taxon	ANI (%)	AAI (%)	dDDH (%)	POCP(%)
*Halomonas salifodinae* IM328	95.9	96.8	66.8	91.9
*Halomonas salifodinae* JCM 14803^ T^	90.79	91.5	42.2	82.1
*Bisbaumannia pacifica* NBRC 102220^ T^	89.9	90.9	39.8	85.1
*Halomonas alkalicola* CICC 11012s^T^	84.2	76.5	24.3	68.9
*Halomonas heilongjiangensis* 9–2 1030^T^	83.6	76.2	24.8	71.3
*Halomonas campaniensis* 5AG^ T^	83.9	75.6	23.7	70.5
*Halomonas mongoliensis* DSM 17332^T^	84.1	75.6	23.9	71.1
*Halomonas flagellata* EGI 63088^ T^	83.5	75.3	23.8	68.5

### Secretory system

The general secretion (Sec) and twin-arginine translocation (Tat) pathways are the most used bacterial secretion systems to transport proteins across the cytoplasmic membrane (Natale et al. 2008). Most proteins transported by the Sec and Tat pathways remain inside the cell, either in the periplasm or the inner membrane. The Sec system can also transport proteins that must stay in the inner membrane through the signal recognition particle (SRP) pathway (Green and Mecsas 2016). The Sec pathway transfers the protein to the periplasmic space before it is folded, and the Tat pathway transfers the folded protein to the periplasm. In contrast, the SRP pathway allows the protein to be folded and transferred simultaneously (Valent et al. 1998). Secretion System Type I is dedicated to transporting digestive enzymes, such as proteases and lipases, as well as adhesins and heme-binding proteins (Green and Mecsas 2016). Meanwhile, the Secretion System Type VI translocates proteins to various recipient cells, including eukaryotic cell targets and, more commonly, other bacteria (Russell et al. 2014). A total of 28 genes related to the bacterial secretion system were found in the genome of A2 (Table S2). We have 11 Sec-SRP genes, three from Tat, one from the Type I secretion system, and 13 from Type VI. The relevance of knowledge of secretory systems lies in the potential genetic modifications of genes associated with these systems to improve the production of value-added biocompounds (Lin et al. 2021).

### Ion transport proteins

In many halophilic microorganisms, potassium absorption and the synthesis of compatible organic solutes control osmotic regulation in conditions of high salinity; K^+^ limitation inhibits growth and adaptation to the saline environment, especially at high salinities, and causes a decrease in intracellular compatible organic solutes (Kraegeloh and Kunte 2002). The Tkr and Pha1 systems regulate K^+^ transport and entry into the cell. The Trk system acts as a transmembrane transport protein, is ATP-dependent, and promotes K^+^ uptake through an electrochemical gradient (Kraegeloh et al. 2005). The Pha1 system functions as a K^+^/H^+^ antiporter with optimal pH involved in potassium transport under slightly alkaline conditions; the Pha1 system can also transport Na^+^ (Yamaguchi et al. 2009). In A2, two genes, *trk*A and *trk*H, belonging to the Trk system, and five Pha1 system genes involved with K^+^ transport were identified (Table S3). Surviving in environments with high salinity is complex; not only does the absorption of K^+^ help keep the osmotic pressure under control, but the expulsion of Na^+^ is essential to maintaining balance. Therefore, precise metabolic systems must be available for the transport of Na^+^. For A2, we found that there are six genes of the Nqr system that encode subunits of NADH: ubiquinone reductase (Na^+^-transporting) and seven genes of the multisubunit sodium/proton antiporter Mrp system (Table S3).

### Secondary metabolites analysis

We used the biosynthetic gene cluster prediction tool by antiSMASH 7.0 to detect Biosynthetic Gene Clusters (BGC) in the genome sequence of A2. The results demonstrate the presence of biosynthetic gene clusters for different types of metabolites mentioned below. Ectoine is a widely used compatible solute by halophilic microorganisms and is highly valued in the industry as an ultra-hydrating agent. Ranthipeptide, an emerging class of natural products belonging to the ribosomally synthesised and posttranslationally modified peptide (RiPP) superfamily, analysis shows that these two ranthipeptides participate in quorum sensing and the control of cellular metabolism (Chen et al. 2020). Resorcinol for the catabolic metabolism of the aromatic compound resorcinol (Yang et al. 2022). Betalactone represents a poorly explored group of secondary metabolites with pharmaceutical potential, many of them with potent bioactivity against bacteria, fungi, or human cancer cell lines. (Džunková et al. 2023; Robinson et al. 2019). Siderophores are synthesised and secreted by many bacteria, yeasts, fungi, and plants for Fe (III) chelation under low iron conditions (Timofeeva et al. 2022).

### Genomic islands

Using IslandViewer 4 software, we identified gene clusters linked to genomic islands in the genome of A2 using the IslandPath-DIMOB and SIGI-HMM prediction methods. The analysis results revealed that A2 has 12 genomic islands with a total of 168 kbp, of which the three largest have a size of 34 kbp, 27 kbp and 19 kbp. Among the genomic islands, there are six genes present (Table S4) that encode proteins directly related to Fe metabolism, TonB-dependent receptor, siderophore-iron reductase FhuF, AraC family transcriptional regulator, ferrioxamine B receptor, iron complex transport system ATP-binding protein and Fe3^+^-hydroxamate ABC transporter permease FhuB, all related to different *Halomonas* species, these genes have great importance in response to alkaline stress (Zhai et al. 2021). Two ISAs1 family transposases from *Halomonas halodenitrificans*, four TypeI-F CRISPR-associated, two associated with proteins, and two associated with helicase and endonuclease from *Halotalea alkalilenta*. In addition, three genes associated with the fimbrial adhesin protein and pilus assembly protein from *Alcanivorax* sp. PN-3.

### Prophage regions

Typically, the prolonged presence of a prophage within a bacterium sometimes leads to the degradation of genetic sequences of the prophage genome, a phenomenon called "phage domestication" (Touchon et al. 2014). Newly integrated phages appear to be inactivated by the host and then eliminate unhelpful genes through point mutations and deletions in genetic regions that are not under selection. Through this process, it can be justified that most prophage sequences found within bacterial genomes are incomplete and do not contain essential genes for interaction with the bacteria (Bobay et al. 2014). An 8.2 Kb extended incomplete prophage region containing ten open reading frames (ORFs) was found at position 31514–39718 with a GC content of 53.46%, within the A2 genome using the PHASTER server. The prophage region was classified as incomplete (< 70 score). This region was confirmed to match the sequences of PHAGE_Entero_mEp460_NC_019716. *Enterobacteriaceae* phage mep460 is a species of dsDNA virus from the class *Caudoviricetes*, bacterial and archaeal viruses with head–tail morphology.

### CRISPR sequence analysis

CRISPR elements are essential for bacterial genomes, providing acquired immunity against viruses and plasmids (Horvath and Barrangou 2010). Several CRISPRs with a unique or different repeat sequence can be found in each strain, but only one of each type is associated with the *cas* genes because the spacers in the CRISPRs are different. The unique sequences or spacers correspond mostly to foreign DNA fragments, i.e. viruses, plasmids, or mobile genetic elements. Several genes called *cas* are associated with CRISPR and are found near them. They perform three different functions of the immune system: adaptation, crRNA maturation, and interference, and their number varies from one type to another. Phylogenetic studies on the CAS protein suggest that CRISPRs are acquired through horizontal transfer. CRISPR loci are transcribed into a pre-crRNA from the leader that acts as a promoter, and then this precursor matures into a small crRNA that plays a role in the selection and destruction of homologous foreign sequences (Couvin et al. 2018; Grissa et al. 2007). In A2, 15 CRISPR regions of 100 to 130 nucleotides in length were located with evidence level one and a CAS-TypeIF *cas* gene cluster region with a level of evidence 4 (Table S5, S6).

### Biotechnological importance genes

Many microorganisms produce natural polyesters such as polyhydroxyalkanoates (PHA) as energy and carbon reserve materials under stressful growth conditions. They can subsequently be degraded by intracellular depolymerases and metabolised as an alternative carbon and energy source (Philip et al. 2007). PHAs have properties like those of conventional petroleum-based plastics, so in addition to their biocompatibility, they are considered materials with high biotechnological potential in industrial applications (Keshavarz and Roy 2010). In A2, the genes *phb*B acetoacetyl-CoA reductase, *phb*C polyhydroxyalkanoate synthase, and *pha*R polyhydroxyalkanoate synthesis repressor PhaR were detected, which participate in the biosynthesis of intracellular PHA.

Alpha-amylase is an enzyme of biotechnological interest because it is used for its antibiofilm properties to control microorganisms. Bacterial biofilms are a significant threat to industries, the environment, and the healthcare sector (Goel et al. 2022). The *amy*A gene encoding alpha-amylase was detected among the unique genes for A2.

Strain A2 also carries multiple genes directly involved in arsenic resistance; these genes have been reported in some species of the *Halomonadaceae* family (Diken et al. 2015; Lin et al. 2012; Wu et al. 2018). The *ars* system of arsenic detoxification is the most studied; its operation begins with the uptake of arsenate by the phosphate transporters of the *pst*ABCS complex (Lin et al. 2012) and the uptake of arsenite by aquaglyceroporins, which transform As (V) into As (III) by arsenate reductases, and the efflux of As (III) by arsenite efflux permeases (Wu et al. 2018). The presence of the genes *ars*A arsenite/tail-anchored protein-transporting ATPase, *ars*B arsenite transporter, *ars*C arsenate reductase, and *ars*H arsenical resistance protein ArsH suggest that A2 is an arsenite-specific efflux and arsenic-reducing prokaryote. These arsenic resistance genes, in addition to previous studies performed in other species, validate the genomic potential of A2 as a candidate organism for bioremediation studies (Diken et al. 2015). Genes of biotechnological importance found in A2 can be seen in supplementary Table S7.

### Pangenome analysis

**Fig. 6:**
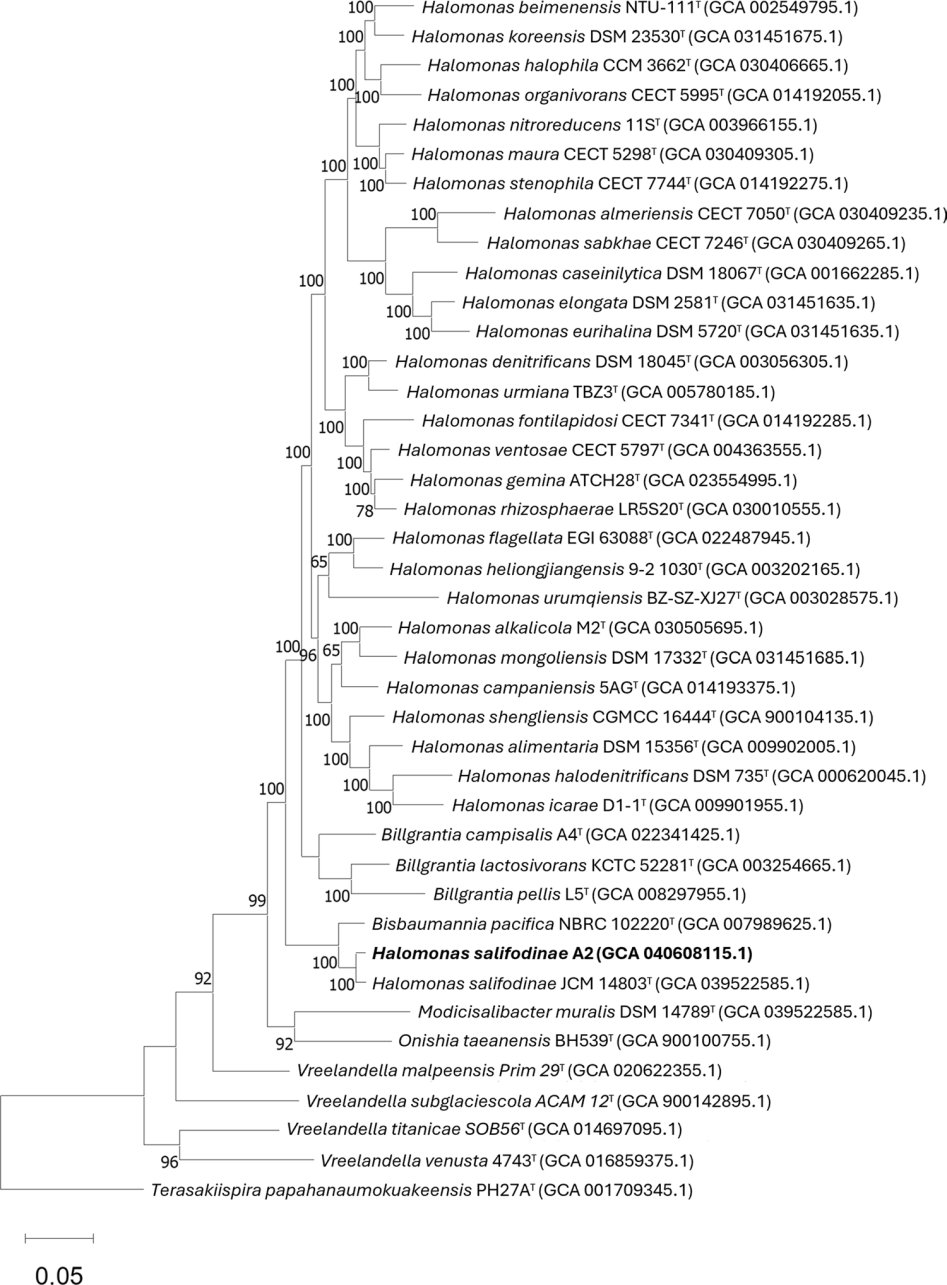
Phylogenetic tree based on core genome sequence showing the phylogenetic position of A2 compared to nearby species. Tree inferred with IQ-TREE server version 2.3.4 to construct the Maximum Likelihood-based on the alignment of 152 core genes from 40 related species using the GTR model with an Ultrafast Bootstrap of 1000

Pangenome analysis was performed using Roary v3.13.0, using GFF3 files generated by Prokka. Consequently, we obtained four different classes of genes belonging to the “core” (95% ≤ strains ≤ 100%), “softcore” (90% ≤ strains < 95%), “shell” (15% ≤ strains < 90%) and “cloud” (0% ≤ strains < 15%). Pangenome analysis was performed using the A2 genome and 100 reference genomes of described *Halomonadaceae* family species found in the NCBI database. Pangenome analysis using Roary revealed 136,122 genes composing the pangenome of A2 and associated species at the 90% threshold. The number of core genes shared by 95–100% of strains was 317, while the cluster of softcore or near-core accessory genes shared by 90% to < 95% of strains was 16. The accessory set of gene clusters widely distributed in the species that form the shell genes were 3,457 genes shared by between 15% and < 95% of the strains. The accessory set of rare genes in the species that form the gene clouds that occur in 0% to < 15% of the strains showed the most significant number of 132,332 genes, also called unique genes. The large number of genes in the cloud implies significant heterogeneity between species (Carpi et al. 2022). The above agrees with what was reported by de la Haba et al. 2023 where the significant genetic variability within the genus is mentioned, which prevents the formation of a monophyletic group, which is why the taxonomy of the genus was reorganised. The results in the phylogenomic tree and pangenome matrix (Fig. 6, S4) constructed from the alignment of 152 core genes from 40 and 15 related species, respectively, show a close relationship between *H. salifodinae* A2 and *H. salifodinae* JCM 14803^ T^; both show a greater close with *B. pacifica* NBRC 102220^ T^ forming a branch separate from the *Halomonas* genus. In addition, the pangenome was constructed using the only three available strains of the genus *Bisbaumannia* was analysed with two only strains of *Halomonas salifodinae* and the A2 strain in which the genes “core” (99% ≤ strains ≤ 100%), “softcore” (95% ≤ strains < 99%), “shell” (15% ≤ strains < 95%) and “cloud” (0% ≤ strains < 15%). This analysis showed a pangenome of 9,399 genes, with 1157 core genes, without softcore genes, 8,242 shell genes and none cloud genes. A large number of core genes and the absence of cloud genes demonstrate high similarity in their genomes (Carpi et al. 2022).

### Singnature genes as phylogenetic evidence

The gene absence-presence matrix calculated by Roary with 100 genomes of species of the *Halomonadaceae* family reveals that A2, *Halomonas salifodinae* JCM 14803^ T^ and *Bisbaumannia pacifica* NBRC 102220^ T^ share 261 signature genes that are not present in any other species of *Halomonadaceae* used in our analysis. These signature genes were corroborated by comparing them with the absence-presence matrix of the pangenome created with the A2, *Bisbaumannia* and *H. salifodinae* strains. This large number of shared unique genes suggests a mutual evolutionary past between both microorganisms (de la Haba et al. 2023) and corroborates that both are more closely related to each other than to the genus *Halomonas* or to any other genus of the *Halomonadaceae* family.

### Unique genes

In A2, 691 unique genes were detected compared with the other 100 *Halomonadaceae* family species and 697 compared to *Halomonas salifodinae* and *Bisbaumannia pacifica* strains. There are 38 unique genes important for adaptability and biotechnological relevance involved in the metabolism of molybdenum, tungsten, copper, iron, antimicrobial resistance systems, biofilm formation, acid resistance, and an entire *bo*_3_-type cytochrome oxidase system. Some of these are described below and in the supplementary material Table S8.

The trace elements molybdenum and tungsten are used by virtually all life forms as binding cofactors in many enzymes (Johnson et al. 1996). Bacterial genes for molybdenum- and tungsten-containing enzymes are often differentially regulated depending on the availability of the metal in the environment (Rajeev et al. 2019). Both Mo and W atoms share several similar chemical characteristics; due to this, there are several strategies to differentiate them and avoid incorrect insertion of the metal into the active site of the enzymes. These elements enter the intracellular medium as soluble oxoanions, MoO_4_^2−^ and WO_4_^2−^, through specific ATP-binding cassette (ABC) transporter systems. Within prokaryotes, these transport systems are divided into three different families: Mod, Wtp, and Tup (Otrelo-Cardoso et al. 2014). The genes *tup*A Tungstate-binding protein TupA and *tup*C Tungstate uptake system ATP-binding protein TupC of the TupABC system were detected; these participate in the cellular uptake of tungsten and belong to the ABC type transport systems (ATP Binding Cassette). The TupA component is a periplasmic protein that binds tungstate anions, which are then transported across the membrane by the TupB component using ATP hydrolysis as an energy source (the reaction catalysed by the ModC component). In addition, the genes related to molybdenum metabolism *mod*A Molybdate-binding protein ModA, *mob*A Molybdenum cofactor guanylyltransferase, *mob*B Molybdopterin-guanine dinucleotide biosynthesis adapter protein, *moe*A_1 Molybdopterin molybdenumtransferase were detected in A2.

Bacteria use flagella for mobility in adverse environments and movement to less hostile sites suitable for adaptation and survival. They are usually formed by multiple protein subunits (He et al. 2023). Flagella assembly is highly ordered and governed by a hierarchical mode of regulation that is tightly controlled by at least 17 operons composed of more than 50 genes (Chevance and Hughes 2008). In A2, nine unique genes were found to be directly involved in synthesising proteins to structure some of the parts of the flagellum, such as the basal body, axial structure, or flagellar filament. Some of these genes are *fli*D1 Flagellar hook-associated protein 2, *fli*F Flagellar M-ring protein, and *flg*A Flagella basal body P-ring formation protein FlgA.

The genes of the cytochrome *bo*_3_ complex were determined as unique genes in A2. Cytochrome *bo*_3_ is encoded by the *cyo*ABCDE operon (Forte et al. 2019). The three gene products of *cyo*A, *cyo*B, and *cyo*C are related to subunits II, I, and III, respectively, of the eukaryotic and prokaryotic *aa*_*3*_-type cytochrome *c* oxidases, and this belongs to the heme-copper oxidase type A-1 superfamily (Sousa et al. 2012). The enzyme generates a proton motive force with high efficiency (H^+^/e^−^ = 2) since it is endowed with proton pumping activity (Puustinen et al. 1991). The cytochrome *bo*_3_ complex predominates in growth conditions where oxygen tension is high (Chepuri et al. 1990). The enzyme consists of four subunits and has three redox cofactors, a low-spin heme *b*, a high-spin heme *o*_3_, and Cu_B_, all located in subunit I. Heme *b* is the primary electron acceptor of ubiquinol, while heme *o*_3_ and Cu_B_ form a binuclear active center where O_2_ chemistry occurs (Melin et al. 2018). As previously thought, Cytochrome *bo*_3_ contains only one ubiquinol binding site located on subunit I instead of two, known as the high-affinity QH site. (Choi et al. 2017).

The Type I protein secretion system *prs*E and *prs*D genes were determined to be unique genes in A2. This system is responsible for the secretion of the EPS-glycanases PlyA and PlyB, two exopolysaccharides (EPS) necessary for biofilm formation (Russo et al. 2006). These enzymes play a crucial role in biofilm formation by cleaving EPS chains and modulating biofilm structure and maturation (Lucke et al. 2020).

A2 would also have the presence of unique genes for a resistance system to acidic environments. The genes *adi*C Arginine/agmatine antiporter and *adi*A biodegradative arginine decarboxylase are reported in *Escherichia coli* as one of three acid resistance systems. The *adi* gene region is organised into two transcriptional units, *adi*AY and *adi*C, which are coordinately regulated but transcribed independently in *E. coli*. The data also illustrate that the AdiA antiporter system and AdiC decarboxylase are designed to function only at high acidity levels, sufficient to damage the cell (Gong et al. 2003). Furthermore, arginine is more than a common amino acid for protein synthesis; it can also be used as the sole nitrogen source for *E. coli* and as a carbon source for many other bacteria. It even functions as a substrate for synthesising polyamines essential for the extreme acid resistance of *E. coli* (Charlier et al. 2019).

In A2 we detected two unique genes, *bep*E and *bep*F, essential in antibiotic resistance and reported in *Brucella suis*. These genes are involved in forming efflux pumps that facilitate the exit of various toxic compounds from the cell. Resistance nodulation-cell division (RND) type efflux pumps are responsible for the multidrug resistance phenotype observed in many clinically relevant species. Furthermore, RND pumps have been implicated in physiological processes, with roles in the virulence mechanisms of several pathogenic bacteria (Martin et al. 2009).

### Genes for the biosynthesis of compatible solutes

Ectoine is synthesised from aspartate through a series of enzymatic reactions. First, L-aspartate-phosphate is synthesised by aspartate kinase (LysC) through ATP-dependent phosphorylation of L-aspartate and catalysed by L-aspartate-beta-semialdehyde dehydrogenase (Asd) through an NADPH-dependent reaction to form L-aspartate-semialdehyde (Schwibbert et al. 2011). Aspartate-semialdehyde is transaminated to 2,4-diaminobutyric acid (DABA); this reaction is catalysed by the DABA transaminase (EctB) encoded in the *ect*B gene. An acetyl group is then transferred to DABA from acetyl-CoA by DABA-N-acetyltransferase (EctA) encoded by the *ect*A gene, synthesising N-acetyl-1,4-diaminobutyric acid (Ono et al. 1999). Finally, ectoine synthase (EctC), encoded by the *ect*C gene, catalyses the cyclic condensation of N-acetyl-l-2,4-diaminobutyric acid, leading to the formation of ectoine (Galinski et al. 1997; Ono et al. 1999). Under certain stress conditions, *Halomonas elongata* and some other halophiles can convert ectoine to 5-hydroxyectoine with ectoine hydroxylase encoded by the *ect*D gene (Bursy et al. 2007; García-Estepa et al. 2006). All genes for ectoine and hydroxyectoine synthesis were found in A2. The classic configuration of these genes in the *Halomonas* genus is maintained in A2, with the *ect*ABC genes arranged continuously in a downstream direction. Still, the *ect*D gene is separated from the other genes in the upstream direction. We compared the A2 *ect*ABC genes configurations with different bacterial species (Fig. S5). Furthermore, ectoine can also be used as a carbon source by using the *doe*ABCDX system. The degradation of ectoine begins by hydrolysis of ectoine to N-αacetyl-1-2,4-diaminobutyric acid by DoeA (ectoine hydrolase) followed by deacetylation of N-α-acetyl-1-2,4-diaminobutyric acid to 1-2,4-diaminobutyric acid by DoeB and a transaminase reaction by DoeD to form L-aspartate semialdehyde. Finally, DoeC can oxidise L-aspartate-semialdehyde to aspartate. DoeX is a Lrp/AsnC family transcriptional regulator. The *doe*ABCDX genes are found in the genome of A2, suggesting that it has the putative ability to degrade ectoine.

The genes for betaine synthesis, *bet*AB, *bet*I, and *bet*T, are present in A2. They synthesise betaine from the precursor choline in a two-step process that involves choline dehydrogenase BetB and betaine aldehyde dehydrogenase BetA; these convert choline to betaine aldehyde and betaine aldehyde to betaine, respectively (Cánovas et al. 1996). The *bet*T gene encodes BetT, a high-affinity choline transporter, and *bet*I encodes BetI, a transcriptional repressor of bet genes (Scholz et al. 2016).

In bacteria, glutamate and glutamine are commonly used as compatible solutes. The glutamate synthesis pathway depends on glutamate dehydrogenase (Gdh) encoded by *gdh*A, while glutamine synthesis falls on glutamine synthetase (Gln) encoded by the *gln*A gene (Kloosterman et al. 2006). The A2 genome contains the genes *gdh*A and *gln*A, which are used to synthesise glutamate and glutamine.

In the genome of A2, we found the *pro*ABC genes involved in proline synthesis. The *pro*B gene encodes glutamate 5-kinase, *pro*A encodes glutamate-5-semialdehyde dehydrogenase, and *pro*C encodes pyrroline-5-carboxylate reductase (Goswami et al. 2022). In addition, other genes related to proline metabolism were found, such as *pro*S encoding a proline-tRNA ligase, *pro*V encoding transport system ATP-binding protein, *pro*W encoding a transport system permease protein, and *pro*X encoding transport system substrate-binding protein all related to glycine/betaine-proline transport, *put*A encodes proline dehydrogenase and *put*P sodium/proline symporter.

## Conclusions

In this study, we performed sequencing and analysis of the genome of strain A2, classified as belonging to the species *Halomonas salifodinae*. The genome has a length of 3.8 Mbp, and the GC content was 67.4%. Genome annotation indicates that pathways directly involved in adapting to hypersaline environments include inorganic ion transport metabolism and amino acid metabolism, with ectoine synthesis being an essential aspect. The different genes found for secretion systems, ion transport, BGC for secondary metabolites, genomic islands, phage sequences, CRISPR sequences and *cas* genes, the genes for compatible solutes and biotechnological importance, and the unique genes found in the genome provide extensive information about the genetic profile of the species that can be used in future research. The pangenome and comparative genomics analysis provided conclusive results about the phylogeny of the species *Halomonas salifodinae*, consistently placing it within a group with *Bisbaumannia pacifica*, the only representative species of the genus *Bisbaumannia* belonging to the *Halomonadaceae* family. The species *Halomonas salifodinae* has been extensively studied for biotechnological purposes (Hu et al. 2021; Yang et al. 2024). The possible reclassification of the *Halomonas salifodinae* species to the *Bisbaumannia* genus opens a new panorama in the taxonomy of the *Halomonadacea*e family since, to date, the *Bisbaumannia* genus, only has one species, *Bisbaumannia pacifica* (de la Haba et al. 2023). Furthermore, due to the difference in genetic content between the two genera, *Bisbaumannia* species have very dissimilar genes compared to those of *Halomonas*. This genetic diversity has a vast potential for using species of the genus *Bisbaumannia* in the biotechnological industry.

The reclassification of *Halomonas salifodinae* to *Bisbaumannia salifodinae* is proposed for future approval following the taxonomic proposal for the genus by de la Haba et al. (2023) with the following taxonomic conclusion:

## Description of *Bisbaumannia salifodinae* comb. nov.

*Bisbaumannia salifodinae sa.li.fo.di’nae.* (L. masc. n. *sal*, salt; L. fem. n. *fodina*, mine; N.L. gen. n. *salifodinae*, of a salt mine)

Basonym: *Halomonas salifodinae* Wang et al. 2008

The description of this species is the same as provided by Wang et al. (2008) for *Halomonas salifodinae*.

Reference genome for the species JCM 14803, accession number GCA_039522585.1. This genome is 4.3 Mb, and its GC content is 66.5%. Genome Geo location name: Salt mine in north-western China. Date: Feb 22, 2023

The type strain is BC7^T^ (= CGMCC 1.6774^ T^ = JCM 14803^ T^)

## Supplementary Information

Below is the link to the electronic supplementary material.

Supplementary file1 (DOCX 4837 KB)

## Data Availability

This Whole Genome Shotgun project has been deposited at DDBJ/ENA/GenBank under the accession. GCA_040608115.1. The genome sequence data could be accessed under the accession number (BioProject PRJNA1114308, Biosample SAMN41481530) in the NCBI database (www.ncbi.nlm.nih. gov). The data from the assembly, annotation and phylogenomic analysis are in https://github.com/A-Leon-Lemus/Bisbaumannia_salifodinae_A2_Genome_Data

## Abbreviations

AAI: Average Amino Acid Identity
ANI: Average Nucleotide Identity
BD: Becton Dickinson
BGC: Biosynthetic gene clusters
BV-BRC: Bacterial and Viral Bioinformatics Resource Center
CDSs: Coding sequences
COG: Clusters of Orthologous Groups
CRISPR: Clustered regularly interspaced short palindromic repeat sequences
DABA: Diaminobutyric acid
dDDH: Digital DNA-DNA Hybridization
EPS: Exopolysaccharides
GGDC: Genome-to-Genome distance calculator
GO: Gene Ontology
GTDB: Genome Taxonomy Database
GTR: General Time-Reversible Model
KEGG: Kyoto Encyclopaedia of Genes and Genomes
MLST: Multilocus Sequence Typing
NCBI: National Center for Biotechnology Information
NJ: Neighbour Joining
NR: Nonredundant Protein Database
OGRIs: Overall Genome Relatedness Indexes
ORFs: Open reading frames
Pfam: Protein Family Database
PHA: Polyhydroxyalkanoates
POCP: Percentage of Conserved Proteins
RND: Resistance nodulation-cell division
Sec: General secretion system
snRNA: Small nuclear RNAs
SRP: Signal recognition particle
Tat: Twin-arginine translocation system
TEM: Transmission electron microscopy
TRF: Tandem repeat finder server
TYGS: Type Strain Genome Server
