# Supplementary material for: Draft genome sequence and comparative genomic analysis of Halomonas salifodinae strain A2 isolated from the Zapotitlán Salinas Valley, Puebla, Mexico
Source: Extremophiles. 2025 Jul 3;29(2):28. doi: 10.1007/s00792-025-01397-z (PMC12226641; doi:10.1007/s00792-025-01397-z)

**Table S1** Genome size and GC percentage of species closely related to strain A2

| ***Related species*** | **Genome length (bp)** | **GC%** |
| --- | --- | --- |
| ***Halomonas salifodinae* A2** | 3,839,280 | 67.4 |
| *Billgrantia campisalis* A4 ^T^ | 4,251,360 | 66.5 |
| *Bisbaumannia pacifica* NBRC ^T^ | 3,794,400 | 67 |
| *Halomonas alimentaria* DSM 15356 ^T^ | 3,278,280 | 66.5 |
| *Halomonas alkalicola* CICC 11012s ^T^ | 3,610,800 | 67.5 |
| *Halomonas almeriensis* CECT 7050 ^T^ | 2,803,980 | 63.5 |
| *Halomonas beimenensis* NTU-111 ^T^ | 4,052,460 | 68.5 |
| *Halomonas campaniensis* 5AG ^T^ | 3,898,440 | 68.5 |
| *Halomonas denitrificants* DSM 18045 ^T^ | 3,751,560 | 68.5 |
| *Halomonas elongata* DSM 2581 ^T^ | 4,061,640 | 63.5 |
| *Halomonas gemina* ATCH28 ^T^ | 3,935,160 | 66.5 |
| *Halomonas huangheensis* BJGMM-B45 ^T^ | 4,757,280 | 58.5 |
| *Halomonas koreensis* DSM 23530 ^T^ | 3,799,500 | 69.5 |
| *Halomonas nitroreducens* 11S ^T^ | 4,329,900 | 67.5 |
| *Halomonas salifodinae* IM328 | 3,952,500 | 67 |
| *Halomonas shengliensis* CGMCC ^T^ | 3,517,980 | 68.5 |
| *Halomonas stenophila* CECT 7744 ^T^ | 4,320,720 | 67.5 |
| *Halomonas urmiana* TBZ3 ^T^ | 3,972,900 | 67 |
| *Halomonas ventosae* CECT 5797 ^T^ | 3,999,420 | 67.5 |
| *Modicisalibacter muralis* DSM 14789 ^T^ | 4,126,920 | 62 |
| *Onishia taeanensis* BH539 ^T^ | 3,741,360 | 62.5 |
| *Vreelandella titanicae* SOB56 ^T^ | 5,279,520 | 54.5 |

**Table S2** Secretion system proteins found in A2

| **Gene ID** | **Identity** | ***Gene Name*** | **NR Description** | **KO ID** | **KO Description** | **Type** |
| --- | --- | --- | --- | --- | --- | --- |
| A2_GM001274 | 82.5 | *secB* | preprotein translocase subunit SecB | K03071 | preprotein translocase subunit SecB | Sec-SRP Secretion System |
| A2_GM003001 | 90.2 | *secE* | preprotein translocase subunit SecE | K03073 | preprotein translocase subunit SecE | Sec-SRP Secretion System |
| A2_GM002968 | 97 | *secY* | preprotein translocase subunit SecY | K03076 | preprotein translocase subunit SecY | Sec-SRP Secretion System |
| A2_GM002784 | 82.2 | *secG* | preprotein translocase subunit SecG | K03075 | preprotein translocase subunit SecG | Sec-SRP Secretion System |
| A2_GM000030 | 90.5 | *ffh* | signal recognition particle protein | K03106 | signal recognition particle subunit SRP54 | Sec-SRP Secretion System |
| A2_GM000057 | 87.7 | *ftsY* | signal recognition particle-docking protein FtsY | K03110 | fused signal recognition particle receptor | Sec-SRP Secretion System |
| A2_GM000121 | 86.9 | *tatC* | twin arginine-targeting protein translocase TatC | K03118 | sec-independent protein translocase protein TatC | Tat Secretion System |
| A2_GM000122 | 66.9 | *tatB* | twin arginine-targeting protein translocase TatB | K03117 | sec-independent protein translocase protein TatB | Tat Secretion System |
| A2_GM000123 | 77.4 | *tatA* | protein translocase TatA | K03116 | sec-independent protein translocase protein TatA | Tat Secretion System |
| A2_GM002073 | 86.7 | *secF* | preprotein translocase subunit SecF | K03074 | preprotein translocase subunit SecF | Sec-SRP Secretion System |
| A2_GM002074 | 84.4 | *secD* | preprotein translocase subunit SecD | K03072 | preprotein translocase subunit SecD | Sec-SRP Secretion System |
| A2_GM002075 | 87.4 | *yajC* | preprotein translocase subunit YajC | K03210 | preprotein translocase subunit YajC | Sec-SRP Secretion System |
| A2_GM002340 | 74.2 | *tolC* | type I secretion protein TolC | K12340 | outer membrane protein | Type I Secretion System |
| A2_GM003271 | 88.3 | *secA* | preprotein translocase subunit SecA | K03070 | preprotein translocase subunit SecA | Sec-SRP Secretion System |
| A2_GM001308 | 82.1 | *yidC* | membrane protein insertase YidC | K03217 | YidC/Oxa1 family membrane protein insertase | Sec-SRP Secretion System |
| A2_GM000454 | 76.6 | *vgrG* | type IV secretion protein Rhs | K11904 | type VI secretion system secreted protein VgrG | Type VI Secretion System |
| A2_GM003475 | 85.8 | *impB* | type VI secretion protein [Halomonas anticariensis] | K11901 | type VI secretion system protein ImpB | Type VI Secretion System |
| A2_GM003476 | 92.1 | *impC* | type VI secretion system protein ImpC [*Halomonas daqingensis*] | K11900 | type VI secretion system protein ImpC | Type VI Secretion System |
| A2_GM003477 | 77.1 | *K11905* | type VI secretion system protein [*Halomonas daqingensis*] | K11905 | type VI secretion system protein | Type VI Secretion System |
| A2_GM003478 | 86.2 | *impG, vasA* | type VI secretion system protein ImpG [*Halomonas pantelleriensis*] | K11896 | type VI secretion system protein ImpG | Type VI Secretion System |
| A2_GM003479 | 81.4 | *impH, vasB* | type VI secretion system protein ImpH [*Halomonas pantelleriensis*] | K11895 | type VI secretion system protein ImpH | Type VI Secretion System |
| A2_GM003481 | 83.7 | *vasD, lip* | type VI secretion lipoprotein [*Halomonas xinjiangensis*] | K11906 | type VI secretion system protein VasD | Type VI Secretion System |
| A2_GM003482 | 86 | *impJ, vasE* | type VI secretion system-associated protein [*Halomonas* sp. KM-1] | K11893 | type VI secretion system protein ImpJ | Type VI Secretion System |
| A2_GM003483 | 77.9 | *impK, ompA, vasF, dotU* | type VI secretion system protein ImpK [*Halomonas pantelleriensis*] | K11892 | type VI secretion system protein ImpK | Type VI Secretion System |
| A2_GM003484 | 83.4 | *vasG, clpV* | ClpV1 family T6SS ATPase [*Halomonas anticariensis]* | K11907 | type VI secretion system protein VasG | Type VI Secretion System |
| A2_GM003486 | 63.9 | *vasI* | type VI secretion system protein VasI [*Halomonas pantelleriensis*] | K11909 | type VI secretion system protein VasI | Type VI Secretion System |
| A2_GM003487 | 63.6 | *vasJ* | type VI secretion system protein VasJ [*Halomonas pantelleriensis*] | K11910 | type VI secretion system protein VasJ | Type VI Secretion System |
| A2_GM003488 | 79.1 | *impL, vasK, icmF* | Fis family transcriptional regulator [*Halomonas* sp. BC04] | K11891 | type VI secretion system protein ImpL | Type VI Secretion System |

**Table S3** Ion transport proteins genes found in A2

| **Gene ID** | **Identity** | ***Gene Name*** | **NR Description** | **KO ID** | **KO Description** |
| --- | --- | --- | --- | --- | --- |
| **Potassium transport** | | | | | |
| A2_GM002044 | 95.4 | *trkA* | trk system potassium uptake protein | K03499 | trk system potassium uptake protein TrkA [*Halomonas pantelleriensis*] |
| A2_GM002043 | 86.6 | *trkH* | trk system potassium uptake protein | K03498 | trk system potassium uptake protein TrkH [*Aidingimonas halophila*] |
| A2_GM000359 | 74 | *phaA* | multisubunit potassium/proton antiporter, PhaA subunit /multisubunit potassium/proton antiporter, PhaB subunit [*Halomonas pantelleriensis*] | K05559 | multicomponent K+:H+ antiporter subunit A |
| A2_GM000358 | 86.7 | *phaC* | NADH-ubiquinone oxidoreductase subunit 4L [*Halomonas chromatireducens*] | K05560 | multicomponent K+:H+ antiporter subunit C |
| A2_GM000356 | 76.8 | *phaE* | K+/H+ antiporter subunit E [*Halomonas* sp. KM-1] | K05562 | multicomponent K+:H+ antiporter subunit E |
| A2_GM000355 | 93.3 | *phaF* | multisubunit potassium/proton antiporter, PhaF subunit [*Halomonas pantelleriensis*] | K05563 | multicomponent K+:H+ antiporter subunit F |
| A2_GM000354 | 82 | *phaG* | K+/H+ antiporter subunit G [*Halomonas* sp. BC04] | K05564 | multicomponent K+:H+ antiporter subunit G |
| **Sodium transport** | | | | | |
| A2_GM003054 | 85.3 | *nqrA* | NADH: ubiquinone reductase (Na(+)-transporting) subunit A [*Halomonas anticariensis*] | K00346 | Na+-transporting NADH:ubiquinone oxidoreductase subunit A |
| A2_GM003053 | 92.1 | *nqrB* | NADH: ubiquinone reductase (Na(+)-transporting) subunit B [*Halomonas* sp. KM-1] | K00347 | Na+-transporting NADH:ubiquinone oxidoreductase subunit B |
| A2_GM003051 | 90.9 | *nqrD* | NADH: ubiquinone reductase (Na(+)-transporting) subunit D [*Halomonas* sp. GFAJ-1] | K00349 | Na+-transporting NADH:ubiquinone oxidoreductase subunit D |
| A2_GM003052 | 87.8 | *nqrC* | NADH: ubiquinone reductase (Na(+)-transporting) subunit C [*Halomonas hydrothermalis*] | K00348 | Na+-transporting NADH:ubiquinone oxidoreductase subunit C |
| A2_GM003050 | 96.1 | *nqrE* | NADH: ubiquinone reductase (Na(+)-transporting) subunit E [*Halomonas* sp. BC04] | K00350 | Na+-transporting NADH:ubiquinone oxidoreductase subunit E |
| A2_GM003049 | 93.5 | *nqrF* | Na+-transporting NADH: ubiquinone oxidoreductase subunit F [*Halomonas daqingensis*] | K00351 | Na+-transporting NADH:ubiquinone oxidoreductase subunit F |
| A2_GM001339 | 85.9 | *ATPF1E, atpC* | F0F1 ATP synthase subunit epsilon [*Halomonas* sp. BC04] | K02114 | F-type H+-transporting ATPase subunit epsilon |
| A2_GM000286 | 94.4 | *nadA* | quinolinate synthetase [*Halomonas pantelleriensis*] | K03517 | quinolinate synthase |
| A2_GM003008 | 80.4 | *nhaC* | transporter, NhaC family (TC 2. A.35) [*Halomonas daqingensis*] | K03315 | Na+:H+ antiporter, NhaC family |
| A2_GM000360 | 82.7 | *mnhA, mrpA* | multisubunit sodium/proton antiporter, MrpA subunit [*Halomonas pantelleriensis*] | K05565 | multicomponent Na+:H+ antiporter subunit A |
| A2_GM000361 | 86.7 | *mnhB, mrpB* | multisubunit sodium/proton antiporter, MrpB subunit (TC 2. A.63. 1) [*Halomonas daqingensis*] | K05566 | multicomponent Na+:H+ antiporter subunit B |
| A2_GM000362 | 94.8 | *mnhC, mrpC* | multisubunit sodium/proton antiporter, MrpC subunit [*Halomonas* *pantelleriensis*] | K05567 | multicomponent Na+:H+ antiporter subunit C |
| A2_GM000363 | 88.5 | *mnhD, mrpD* | multisubunit sodium/proton antiporter, MrpD subunit [*Halomonas pantelleriensis*] | K05568 | multicomponent Na+:H+ antiporter subunit D |
| A2_GM000364 | 89.8 | *mnhE, mrpE* | multisubunit sodium/proton antiporter, MrpE subunit [*Halomonas* *daqingensis*] | K05569 | multicomponent Na+:H+ antiporter subunit E |
| A2_GM000365 | 89.5 | *mnhF, mrpF* | multiple resistance and pH regulation protein F [*Halomonas* sp.] | K05570 | multicomponent Na+:H+ antiporter subunit F |
| A2_GM000366 | 88.8 | *mnhG, mrpG* | Multicomponent Na+: H+ antiporter subunit G [*Halomonas* sp. R57-5] | K05571 | multicomponent Na+:H+ antiporter subunit G |

**Table S4** Genes found in the genomic islands detected in the genome of A2

| **Island start** | **Island end** | **Length** | **Method** | **Gene ID** | **Locus** | **Gene start** | **Gene end** | **Strand** | **Product** |
| --- | --- | --- | --- | --- | --- | --- | --- | --- | --- |
| 299462 | 326252 | 26790 | IslandPath-DIMOB | A2_GM002623 | A2_GM002623 | 299462 | 300163 | 1 | GMP synthase (glutamine-hydrolysing) [*Halomonas daqiaonensis*] |
| 299462 | 326252 | 26790 | IslandPath-DIMOB | A2_GM003435 | A2_GM003435 | 301681 | 303798 | 1 | type IV secretion protein Rhs [*Halomonas* sp. BC04] |
| 299462 | 326252 | 26790 | IslandPath-DIMOB | A2_GM003434 | A2_GM003434 | 303795 | 304661 | 1 | hypothetical protein [*Halomonas* sp.] |
| 299462 | 326252 | 26790 | IslandPath-DIMOB | A2_GM003433 | A2_GM003433 | 304658 | 308194 | 1 | hypothetical protein [*Halomonas xinjiangensis*] |
| 299462 | 326252 | 26790 | IslandPath-DIMOB | A2_GM003432 | A2_GM003432 | 308430 | 309176 | 1 | hypothetical protein [*Halomonas anticariensis*] |
| 299462 | 326252 | 26790 | IslandPath-DIMOB | A2_GM003431 | A2_GM003431 | 309410 | 311227 | 1 | phage-related baseplate assembly protein [*Halomonas elongata*] |
| 299462 | 326252 | 26790 | IslandPath-DIMOB | A2_GM003430 | A2_GM003430 | 311224 | 312039 | 1 | hypothetical protein [*Halomonas* sp.] |
| 299462 | 326252 | 26790 | IslandPath-DIMOB | A2_GM003429 | A2_GM003429 | 312135 | 315293 | 1 | hypothetical protein [*Halomonas daqingensis*, SAMN04487953_13135] |
| 299462 | 326252 | 26790 | IslandPath-DIMOB | A2_GM003428 | A2_GM003428 | 315328 | 316278 | 1 | hypothetical protein [*Halomonas* sp. BC04, Q427_02530] |
| 299462 | 326252 | 26790 | IslandPath-DIMOB | A2_GM003427 | A2_GM003427 | 316297 | 317274 | 1 | hypothetical protein [*Halomonas* sp.] |
| 299462 | 326252 | 26790 | IslandPath-DIMOB | A2_GM003426 | A2_GM003426 | 317293 | 318264 | 1 | hypothetical protein [*Halomonas* sp.] |
| 299462 | 326252 | 26790 | IslandPath-DIMOB | A2_GM003425 | A2_GM003425 | 318271 | 319251 | 1 | hypothetical protein [*Halomonas* sp. HL-93, GA0071314_1508] |
| 299462 | 326252 | 26790 | IslandPath-DIMOB | A2_GM003424 | A2_GM003424 | 319251 | 320249 | 1 | hypothetical protein [*Halomonas* sp. SUBG004, DK37_19800] |
| 299462 | 326252 | 26790 | IslandPath-DIMOB | A2_GM003423 | A2_GM003423 | 320249 | 321247 | 1 | hypothetical protein [*Halomonas* sp. BC04, Q427_02530] |
| 299462 | 326252 | 26790 | IslandPath-DIMOB | A2_GM003422 | A2_GM003422 | 322307 | 324502 | -1 | hypothetical protein [*Halomonas* anticariensis] |
| 299462 | 326252 | 26790 | IslandPath-DIMOB | A2_GM003421 | A2_GM003421 | 324611 | 325063 | 1 | Cd(II)/Pb(II)-responsive transcriptional regulator [*Halomonas anticariensis*] |
| 299462 | 326252 | 26790 | IslandPath-DIMOB | A2_GM003420 | A2_GM003420 | 325107 | 326252 | -1 | beta-aspartyl-peptidase [*Halomonas smyrnensis*] |
| 1805657 | 1813658 | 8001 | IslandPath-DIMOB | A2_GM002834 | A2_GM002834 | 1805657 | 1806385 | 1 | hypothetical protein [*Halomonas anticariensis*] |
| 1805657 | 1813658 | 8001 | IslandPath-DIMOB | A2_GM002835 | A2_GM002835 | 1806372 | 1807292 | 1 | integrase/recombinase XerC [*Halomonas taeanensis*] |
| 1805657 | 1813658 | 8001 | IslandPath-DIMOB | A2_GM002836 | A2_GM002836 | 1807289 | 1808035 | 1 | putative hydrolase of the HAD superfamily [*Halomonas taeanensis*] |
| 1805657 | 1813658 | 8001 | IslandPath-DIMOB | A2_GM002837 | A2_GM002837 | 1808042 | 1808650 | -1 | putative GTP-binding protein EngB [*Halomonas chromatireducens*] |
| 1805657 | 1813658 | 8001 | IslandPath-DIMOB | A2_GM002838 | A2_GM002838 | 1808875 | 1809489 | 1 | cytochrome c [*Halomonas anticariensis*] |
| 1805657 | 1813658 | 8001 | IslandPath-DIMOB | A2_GM002839 | A2_GM002839 | 1809654 | 1810292 | 1 | thiol: disulfide interchange protein DsbA [*Halomonas daqingensis*] |
| 1805657 | 1813658 | 8001 | IslandPath-DIMOB | A2_GM002840 | A2_GM002840 | 1810297 | 1811118 | 1 | Metal-dependent hydrolase, endonuclease/exonuclease/phosphatase family [*Halomonas daqiaonensis*] |
| 1805657 | 1813658 | 8001 | IslandPath-DIMOB | A2_GM002841 | A2_GM002841 | 1811238 | 1811762 | 1 | Tripartite ATP-independent periplasmic transporter, DctQ component [*Halomonas chromatireducens*] |
| 1805657 | 1813658 | 8001 | IslandPath-DIMOB | A2_GM002842 | A2_GM002842 | 1811762 | 1813165 | 1 | TRAP transporter large transmembrane protein [*Halomonas* sp. GFAJ-1] |
| 1805657 | 1813658 | 8001 | IslandPath-DIMOB | A2_GM002843 | A2_GM002843 | 1813221 | 1813658 | -1 | Inner membrane protein YqaA [*Halomonas chromatireducens*] |
| 1890972 | 1900311 | 9339 | IslandPath-DIMOB | A2_GM002131 | A2_GM002131 | 1890972 | 1891322 | -1 | hypothetical protein [*Lacimicrobium alkaliphilum*] |
| 1890972 | 1900311 | 9339 | IslandPath-DIMOB | A2_GM002132 | A2_GM002132 | 1891407 | 1891835 | -1 | acetyltransferase (GNAT) family protein [*Halomonas elongata* DSM 2581] |
| 1890972 | 1900311 | 9339 | IslandPath-DIMOB | A2_GM002133 | A2_GM002133 | 1891894 | 1892610 | -1 | alpha/beta hydrolase [*Thioalkalivibrio nitratireducens*] |
| 1890972 | 1900311 | 9339 | IslandPath-DIMOB | A2_GM002134 | A2_GM002134 | 1893073 | 1893519 | -1 | hypothetical protein |
| 1890972 | 1900311 | 9339 | IslandPath-DIMOB | A2_GM002135 | A2_GM002135 | 1893576 | 1894394 | -1 | Restriction endonuclease [*Halomonas arcis*] |
| 1890972 | 1900311 | 9339 | IslandPath-DIMOB | A2_GM002136 | A2_GM002136 | 1898461 | 1898877 | 1 | site-specific tyrosine recombinase XerC [*Halomonas chromatireducens*] |
| 1890972 | 1900311 | 9339 | IslandPath-DIMOB | A2_GM002137 | A2_GM002137 | 1898874 | 1899044 | 1 | hypothetical protein |
| 1890972 | 1900311 | 9339 | IslandPath-DIMOB | A2_GM002138 | A2_GM002138 | 1899511 | 1900311 | -1 | indole-3-glycerol phosphate synthase [*Halomonas pantelleriensis*] |
| 2073859 | 2086522 | 12663 | IslandPath-DIMOB | A2_GM002314 | A2_GM002314 | 2073859 | 2074785 | -1 | electron transfer flavoprotein subunit beta [*Halomonas smyrnensis*] |
| 2073859 | 2086522 | 12663 | IslandPath-DIMOB | A2_GM002315 | A2_GM002315 | 2074782 | 2075534 | -1 | electron transfer flavoprotein beta subunit [*Halomonas shengliensis*] |
| 2073859 | 2086522 | 12663 | IslandPath-DIMOB | A2_GM000842 | A2_GM000842 | 2078375 | 2078713 | -1 | hypothetical protein |
| 2073859 | 2086522 | 12663 | IslandPath-DIMOB | A2_GM000843 | A2_GM000843 | 2078955 | 2079149 | 1 | addiction module toxin, HicA family [*Halomonas elongata*] |
| 2073859 | 2086522 | 12663 | IslandPath-DIMOB | A2_GM000844 | A2_GM000844 | 2079185 | 2079598 | 1 | phage-like protein [*Halomonas* sp. PR-M31] |
| 2073859 | 2086522 | 12663 | IslandPath-DIMOB | A2_GM000845 | A2_GM000845 | 2079679 | 2080080 | 1 | hypothetical protein [*Chromohalobacter japonicus*] |
| 2073859 | 2086522 | 12663 | IslandPath-DIMOB | A2_GM000846 | A2_GM000846 | 2080100 | 2080507 | 1 | hypothetical protein [*Chromohalobacter japonicus*] |
| 2073859 | 2086522 | 12663 | IslandPath-DIMOB | A2_GM000847 | A2_GM000847 | 2080536 | 2080727 | -1 | hypothetical protein [*Halomonas shengliensis*, SAMN04487957_102215] |
| 2073859 | 2086522 | 12663 | IslandPath-DIMOB | A2_GM000848 | A2_GM000848 | 2080828 | 2084505 | -1 | sulfotransferase [*Halomonas* sp. KM-1] |
| 2073859 | 2086522 | 12663 | IslandPath-DIMOB | A2_GM000849 | A2_GM000849 | 2085209 | 2086522 | -1 | flagellin [*Halomonas* sp. GFAJ-1] |
| 2339846 | 2374273 | 34427 | IslandPath-DIMOB | A2_GM001085 | A2_GM001085 | 2338089 | 2339852 | 1 | long-chain acyl-CoA synthetase [*Halomonas pantelleriensis*] |
| 2339846 | 2374273 | 34427 | IslandPath-DIMOB | A2_GM001086 | A2_GM001086 | 2339846 | 2340757 | 1 | bile acid: Na+ symporter, BASS family [*Halomonas pantelleriensis*] |
| 2339846 | 2374273 | 34427 | IslandPath-DIMOB | A2_GM001087 | A2_GM001087 | 2340823 | 2341851 | -1 | 5-methyltetrahydropteroyltriglutamate-- homocysteine methyltransferase [*Halomonas halodenitrificans*] |
| 2339846 | 2374273 | 34427 | IslandPath-DIMOB | A2_GM001088 | A2_GM001088 | 2341877 | 2342860 | -1 | hypothetical protein [*Halomonas halodenitrificans*] |
| 2339846 | 2374273 | 34427 | IslandPath-DIMOB | A2_GM001089 | A2_GM001089 | 2343255 | 2345141 | 1 | methyl-accepting chemotaxis protein *[Halomonas gudaonensis*] |
| 2339846 | 2374273 | 34427 | IslandPath-DIMOB | A2_GM001090 | A2_GM001090 | 2346331 | 2346669 | 1 | integrase catalytic subunit [*Alcanivorax pacificus* W11-5] |
| 2339846 | 2374273 | 34427 | IslandPath-DIMOB | A2_GM001091 | A2_GM001091 | 2348102 | 2348206 | 1 | hypothetical protein |
| 2339846 | 2374273 | 34427 | IslandPath-DIMOB | A2_GM001092 | A2_GM001092 | 2348716 | 2351940 | -1 | type III restriction enzyme, res subunit [*Halomonas huangheensis*] |
| 2339846 | 2374273 | 34427 | IslandPath-DIMOB | A2_GM001093 | A2_GM001093 | 2352134 | 2353285 | 1 | hypothetical protein [*Halomonas arcis*, SAMN04487951_101360] |
| 2339846 | 2374273 | 34427 | IslandPath-DIMOB | A2_GM001094 | A2_GM001094 | 2353394 | 2354707 | -1 | hypothetical protein [*Halomonas* sp. PR-M31] |
| 2339846 | 2374273 | 34427 | IslandPath-DIMOB | A2_GM001095 | A2_GM001095 | 2354704 | 2355684 | -1 | hypothetical protein [*Halomonas huangheensis]* |
| 2339846 | 2374273 | 34427 | IslandPath-DIMOB | A2_GM001096 | A2_GM001096 | 2355821 | 2357755 | -1 | N-6 DNA methylase [*Halomonas* sp. PBN3] |
| 2339846 | 2374273 | 34427 | IslandPath-DIMOB | A2_GM001097 | A2_GM001097 | 2358190 | 2358795 | 1 | hypothetical protein [*Halomonas taeanensis*, SAMN05216571_102414] |
| 2339846 | 2374273 | 34427 | IslandPath-DIMOB | A2_GM001098 | A2_GM001098 | 2359072 | 2361189 | 1 | TonB-denpendent receptor [*Halomonas anticariensis* FP35 = DSM 16096] |
| 2339846 | 2374273 | 34427 | IslandPath-DIMOB | A2_GM001099 | A2_GM001099 | 2361197 | 2361925 | 1 | siderophore-iron reductase FhuF [*Halomonas salina*] |
| 2339846 | 2374273 | 34427 | IslandPath-DIMOB | A2_GM001100 | A2_GM001100 | 2361941 | 2362876 | 1 | AraC family transcriptional regulator [*Halomonas alkaliantarctica*] |
| 2339846 | 2374273 | 34427 | IslandPath-DIMOB | A2_GM001101 | A2_GM001101 | 2362983 | 2365058 | 1 | ferrioxamine B receptor [*Halomonas alkaliantarctica*] |
| 2339846 | 2374273 | 34427 | IslandPath-DIMOB | A2_GM001102 | A2_GM001102 | 2365131 | 2366930 | -1 | ATP-binding cassette, subfamily B [*Halomonas daqingensis*] |
| 2339846 | 2374273 | 34427 | IslandPath-DIMOB | A2_GM001103 | A2_GM001103 | 2367066 | 2367833 | 1 | iron complex transport system ATP-binding protein [*Halomonas shengliensis*] |
| 2339846 | 2374273 | 34427 | IslandPath-DIMOB | A2_GM001104 | A2_GM001104 | 2367837 | 2368718 | 1 | hypothetical protein [*Halomonas* sp. PBN3, Q671_06025] |
| 2339846 | 2374273 | 34427 | IslandPath-DIMOB | A2_GM001105 | A2_GM001105 | 2368715 | 2370715 | 1 | Fe3+-hydroxamate ABC transporter permease FhuB [*Halomonas smyrnensis*] |
| 2339846 | 2374273 | 34427 | IslandPath-DIMOB | A2_GM001106 | A2_GM001106 | 2370795 | 2371154 | -1 | RidA/YER057c/UK114 family protein [*Halomonas elongata*] |
| 2339846 | 2374273 | 34427 | IslandPath-DIMOB | A2_GM001107 | A2_GM001107 | 2371234 | 2371587 | -1 | molecular chaperone DnaK [*Halomonas* sp. BC04] |
| 2339846 | 2374273 | 34427 | IslandPath-DIMOB | A2_GM001108 | A2_GM001108 | 2371713 | 2372906 | 1 | Predicted arabinose efflux permease, MFS family [*Halomonas gudaonensis]* |
| 2339846 | 2374273 | 34427 | IslandPath-DIMOB | A2_GM001109 | A2_GM001109 | 2372967 | 2373806 | -1 | Nucleotide-binding universal stress protein, UspA family [*Halomonas caseinilytica*] |
| 2339846 | 2374273 | 34427 | IslandPath-DIMOB | A2_GM001110 | A2_GM001110 | 2373926 | 2374273 | -1 | antibiotic biosynthesis monooxygenase [*Halomonas halodenitrificans*] |
| 3439138 | 3444592 | 5454 | IslandPath-DIMOB | A2_GM003021 | A2_GM003021 | 3439138 | 3439380 | 1 | MULTISPECIES: antitoxin [*Pseudomonas \| Pseudomonas* sp.] |
| 3439138 | 3444592 | 5454 | IslandPath-DIMOB | A2_GM003020 | A2_GM003020 | 3439380 | 3439781 | 1 | plasmid maintenance protein [*Pseudomonas* sp. 5] |
| 3439138 | 3444592 | 5454 | IslandPath-DIMOB | A2_GM003019 | A2_GM003019 | 3440118 | 3440519 | 1 | Predicted nuclease of the RNAse H fold, HicB family [*Ectothiorhodospira magna*] |
| 3439138 | 3444592 | 5454 | IslandPath-DIMOB | A2_GM003018 | A2_GM003018 | 3440559 | 3441515 | 1 | putative transposase [*Marinobacter* sp. ELB17] |
| 3439138 | 3444592 | 5454 | IslandPath-DIMOB | A2_GM003017 | A2_GM003017 | 3441789 | 3442496 | 1 | hypothetical protein [*Halomonas* sp.] |
| 3439138 | 3444592 | 5454 | IslandPath-DIMOB | A2_GM003016 | A2_GM003016 | 3442738 | 3443130 | 1 | CoA-binding protein [*Azovibrio restrictus]* |
| 3439138 | 3444592 | 5454 | IslandPath-DIMOB | A2_GM003015 | A2_GM003015 | 3443300 | 3444592 | -1 | nucleobase: cation symporter-2, NCS2 family [*Halomonas muralis*] |
| 3482518 | 3502373 | 19855 | IslandPath-DIMOB | A2_GM003383 | A2_GM003383 | 3482518 | 3484080 | -1 | AAA-ATPase-like protein [*Halomonas* sp. KO116] |
| 3482518 | 3502373 | 19855 | IslandPath-DIMOB | A2_GM003384 | A2_GM003384 | 3484416 | 3485528 | -1 | histone deacetylase-like amidohydrolase [*Halomonas elongata*] |
| 3482518 | 3502373 | 19855 | IslandPath-DIMOB | A2_GM003385 | A2_GM003385 | 3485556 | 3487013 | -1 | Na+/H+ antiporter family protein [*Halomonas elongata*] |
| 3482518 | 3502373 | 19855 | IslandPath-DIMOB | A2_GM003386 | A2_GM003386 | 3487365 | 3488441 | 1 | dTDP-glucose 4, 6-dehydratase [*Halomonas salina*] |
| 3482518 | 3502373 | 19855 | IslandPath-DIMOB | A2_GM003387 | A2_GM003387 | 3488508 | 3489386 | 1 | glucose-1-phosphate thymidylyltransferase [*Halomonas daqiaonensis*] |
| 3482518 | 3502373 | 19855 | IslandPath-DIMOB | A2_GM003388 | A2_GM003388 | 3492223 | 3493578 | 1 | polysaccharide transporter, PST family/teichuronic acid exporter [*Pseudomonas salegens*] |
| 3482518 | 3502373 | 19855 | IslandPath-DIMOB | A2_GM003389 | A2_GM003389 | 3495470 | 3495649 | 1 | ISAs1 family transposase, partial [*Halomonas halodenitrificans*] |
| 3482518 | 3502373 | 19855 | IslandPath-DIMOB | A2_GM003390 | A2_GM003390 | 3495701 | 3496600 | 1 | ISAs1 family transposase [*Halomonas halodenitrificans*] |
| 3482518 | 3502373 | 19855 | IslandPath-DIMOB | A2_GM003391 | A2_GM003391 | 3498985 | 3499629 | 1 | glycosyl transferase [*Arcobacter skirrowii]* |
| 3482518 | 3502373 | 19855 | IslandPath-DIMOB | A2_GM003392 | A2_GM003392 | 3499648 | 3500574 | 1 | nucleoside-diphosphate sugar epimerase [*Halomonas lutea*] |
| 3482518 | 3502373 | 19855 | IslandPath-DIMOB | A2_GM003393 | A2_GM003393 | 3500571 | 3501125 | 1 | lipid carrier--UDP-N-acetylgalactosaminyltransferase [*Halomonas salina*] |
| 3482518 | 3502373 | 19855 | IslandPath-DIMOB | A2_GM003394 | A2_GM003394 | 3501207 | 3502373 | 1 | UDP-glucose 6-dehydrogenase [*Halomonas salina*] |
| 146618 | 154688 | 8070 | SIGI-HMM | A2_GM002482 | A2_GM002482 | 146618 | 147130 | 1 | hypothetical protein SAMN05443545_1154, partial [*Aidingimonas halophila*] |
| 146618 | 154688 | 8070 | SIGI-HMM | A2_GM002483 | A2_GM002483 | 147111 | 147800 | 1 | hypothetical protein [*Halomonas elongata*, A8U91_00338] |
| 146618 | 154688 | 8070 | SIGI-HMM | A2_GM002484 | A2_GM002484 | 148066 | 148605 | 1 | hypothetical protein SAMN05192555_11188, partial [*Halomonas pantelleriensis*] |
| 146618 | 154688 | 8070 | SIGI-HMM | A2_GM002485 | A2_GM002485 | 148670 | 149281 | 1 | hypothetical protein [*Halomonas xinjiangensis*] |
| 146618 | 154688 | 8070 | SIGI-HMM | A2_GM002486 | A2_GM002486 | 150145 | 150741 | 1 | hypothetical protein [*Halomonas elongata*, A8U91_00338] |
| 146618 | 154688 | 8070 | SIGI-HMM | A2_GM002487 | A2_GM002487 | 151374 | 152045 | 1 | hypothetical protein [*Halomonas anticariensis*] |
| 146618 | 154688 | 8070 | SIGI-HMM | A2_GM002488 | A2_GM002488 | 152049 | 153047 | 1 | hypothetical protein [*Halomonas daqingensis*, SAMN04487953_13132] |
| 146618 | 154688 | 8070 | SIGI-HMM | A2_GM002489 | A2_GM002489 | 153053 | 154048 | 1 | hypothetical protein [*Halomonas* sp. KM-1] |
| 146618 | 154688 | 8070 | SIGI-HMM | A2_GM002490 | A2_GM002490 | 154566 | 154688 | -1 | hypothetical protein |
| 303795 | 309176 | 5381 | SIGI-HMM | A2_GM003435 | A2_GM003435 | 301681 | 303798 | 1 | type IV secretion protein Rhs [*Halomonas* sp. BC04] |
| 303795 | 309176 | 5381 | SIGI-HMM | A2_GM003434 | A2_GM003434 | 303795 | 304661 | 1 | hypothetical protein [*Halomonas* sp.] |
| 303795 | 309176 | 5381 | SIGI-HMM | A2_GM003433 | A2_GM003433 | 304658 | 308194 | 1 | hypothetical protein [*Halomonas* *xinjiangensis*] |
| 303795 | 309176 | 5381 | SIGI-HMM | A2_GM003432 | A2_GM003432 | 308430 | 309176 | 1 | hypothetical protein [*Halomonas anticariensis*] |
| 315328 | 321247 | 5919 | SIGI-HMM | A2_GM003428 | A2_GM003428 | 315328 | 316278 | 1 | hypothetical protein [*Halomonas* sp. BC04, Q427_02530] |
| 315328 | 321247 | 5919 | SIGI-HMM | A2_GM003427 | A2_GM003427 | 316297 | 317274 | 1 | hypothetical protein [*Halomonas* sp.] |
| 315328 | 321247 | 5919 | SIGI-HMM | A2_GM003426 | A2_GM003426 | 317293 | 318264 | 1 | hypothetical protein [*Halomonas* sp*.]* |
| 315328 | 321247 | 5919 | SIGI-HMM | A2_GM003425 | A2_GM003425 | 318271 | 319251 | 1 | hypothetical protein [*Halomonas* sp. HL-93, GA0071314_1508] |
| 315328 | 321247 | 5919 | SIGI-HMM | A2_GM003424 | A2_GM003424 | 319251 | 320249 | 1 | hypothetical protein [*Halomonas* sp. SUBG004, DK37_19800] |
| 315328 | 321247 | 5919 | SIGI-HMM | A2_GM003423 | A2_GM003423 | 320249 | 321247 | 1 | hypothetical protein [*Halomonas* sp. BC04, Q427_02530] |
| 1720614 | 1724781 | 4167 | SIGI-HMM | A2_GM002626 | A2_GM002626 | 1720614 | 1722176 | -1 | hypothetical protein [*Halomonas* *chromatireducens*, LOKO_01754] |
| 1720614 | 1724781 | 4167 | SIGI-HMM | A2_GM002625 | A2_GM002625 | 1723439 | 1723927 | -1 | hypothetical protein [Halomonas *chromatireducens*] |
| 1720614 | 1724781 | 4167 | SIGI-HMM | A2_GM002624 | A2_GM002624 | 1723927 | 1724781 | -1 | hypothetical protein [*Halomonas xinjiangensis*] |
| 1893073 | 1900311 | 7238 | SIGI-HMM | A2_GM002134 | A2_GM002134 | 1893073 | 1893519 | -1 | hypothetical protein |
| 1893073 | 1900311 | 7238 | SIGI-HMM | A2_GM002135 | A2_GM002135 | 1893576 | 1894394 | -1 | Restriction endonuclease [*Halomonas arcis*] |
| 1893073 | 1900311 | 7238 | SIGI-HMM | A2_GM002136 | A2_GM002136 | 1898461 | 1898877 | 1 | site-specific tyrosine recombinase XerC [*Halomonas chromatireducens*] |
| 1893073 | 1900311 | 7238 | SIGI-HMM | A2_GM002137 | A2_GM002137 | 1898874 | 1899044 | 1 | hypothetical protein |
| 1893073 | 1900311 | 7238 | SIGI-HMM | A2_GM002138 | A2_GM002138 | 1899511 | 1900311 | -1 | indole-3-glycerol phosphate synthase [*Halomonas pantelleriensis*] |
| 2142222 | 2153145 | 10923 | SIGI-HMM | A2_GM000907 | A2_GM000907 | 2142222 | 2142485 | 1 | hypothetical protein |
| 2142222 | 2153145 | 10923 | SIGI-HMM | A2_GM000908 | A2_GM000908 | 2142981 | 2143436 | -1 | very short patch repair endonuclease [*Halomonas* sp. PBN3] |
| 2142222 | 2153145 | 10923 | SIGI-HMM | A2_GM000909 | A2_GM000909 | 2143630 | 2143947 | -1 | hypothetical protein |
| 2142222 | 2153145 | 10923 | SIGI-HMM | A2_GM000910 | A2_GM000910 | 2144005 | 2145159 | 1 | DNA-cytosine methyltransferase [*Halomonas elongata* DSM 2581] |
| 2142222 | 2153145 | 10923 | SIGI-HMM | A2_GM000911 | A2_GM000911 | 2145217 | 2146041 | -1 | hypothetical protein [*Halomonas elongata*] |
| 2142222 | 2153145 | 10923 | SIGI-HMM | A2_GM000912 | A2_GM000912 | 2146038 | 2146943 | -1 | hypothetical protein [*Halomonas elongata*] |
| 2142222 | 2153145 | 10923 | SIGI-HMM | A2_GM000913 | A2_GM000913 | 2146940 | 2147212 | -1 | hypothetical protein |
| 2142222 | 2153145 | 10923 | SIGI-HMM | A2_GM000914 | A2_GM000914 | 2147801 | 2148517 | 1 | hypothetical protein |
| 2142222 | 2153145 | 10923 | SIGI-HMM | A2_GM000915 | A2_GM000915 | 2148787 | 2149797 | -1 | hypothetical protein [*Halomonas elongata*] |
| 2142222 | 2153145 | 10923 | SIGI-HMM | A2_GM000916 | A2_GM000916 | 2149790 | 2152432 | -1 | hypothetical protein [*Halomonas elongata* DSM 2581, HELO_4400] |
| 2142222 | 2153145 | 10923 | SIGI-HMM | A2_GM000917 | A2_GM000917 | 2152598 | 2152969 | -1 | PIN domain-containing protein [*Halomonas salina*] |
| 2142222 | 2153145 | 10923 | SIGI-HMM | A2_GM000918 | A2_GM000918 | 2152969 | 2153145 | -1 | hypothetical protein [*Halomonas* sp. BC04, Q427_16230] |
| 2663668 | 2671689 | 8021 | SIGI-HMM | A2_GM000302 | A2_GM000302 | 2662955 | 2663671 | 1 | tRNA-Thr(GGU) m(6)t(6)A37 methyltransferase TsaA[*Halomonas pantelleriensis*] |
| 2663668 | 2671689 | 8021 | SIGI-HMM | A2_GM000301 | A2_GM000301 | 2663668 | 2665965 | -1 | hypothetical protein [*Alcanivorax* sp.] |
| 2663668 | 2671689 | 8021 | SIGI-HMM | A2_GM000300 | A2_GM000300 | 2665992 | 2667017 | -1 | hypothetical protein [*Alcanivorax* sp. 43B_GOM-46m] |
| 2663668 | 2671689 | 8021 | SIGI-HMM | A2_GM000299 | A2_GM000299 | 2667333 | 2668310 | -1 | fimbrial adhesin [*Alcanivorax dieselole*i] |
| 2663668 | 2671689 | 8021 | SIGI-HMM | A2_GM000298 | A2_GM000298 | 2668342 | 2670726 | -1 | fimbrial protein [*Alcanivorax* sp. PN-3] |
| 2663668 | 2671689 | 8021 | SIGI-HMM | A2_GM000297 | A2_GM000297 | 2670961 | 2671689 | -1 | MULTISPECIES: pilus assembly protein [*Alcanivorax*\|*Alcanivorax* sp.] |
| 3495470 | 3499629 | 4159 | SIGI-HMM | A2_GM003389 | A2_GM003389 | 3495470 | 3495649 | 1 | ISAs1 family transposase, partial [*Halomonas halodenitrificans*] |
| 3495470 | 3499629 | 4159 | SIGI-HMM | A2_GM003390 | A2_GM003390 | 3495701 | 3496600 | 1 | ISAs1 family transposase [*Halomonas halodenitrificans*] |
| 3495470 | 3499629 | 4159 | SIGI-HMM | A2_GM003391 | A2_GM003391 | 3498985 | 3499629 | 1 | glycosyl transferase [*Arcobacter skirrowii*] |
| 3587877 | 3594001 | 6124 | SIGI-HMM | A2_GM001468 | A2_GM001468 | 3587877 | 3588854 | -1 | type I-F CRISPR-associated protein Csy2 [*Halotalea alkalilenta*] |
| 3587877 | 3594001 | 6124 | SIGI-HMM | A2_GM001467 | A2_GM001467 | 3588851 | 3590170 | -1 | type I-F CRISPR-associated protein Csy1 [*Halotalea alkalilenta*] |
| 3587877 | 3594001 | 6124 | SIGI-HMM | A2_GM001466 | A2_GM001466 | 3590660 | 3594001 | -1 | type I-F CRISPR-associated helicase Cas3 [*Halotalea alkalilenta*] |
| 3587877 | 3594001 | 6124 | SIGI-HMM | A2_GM001465 | A2_GM001465 | 3593998 | 3594972 | -1 | subtype I-F CRISPR-associated endonuclease Cas1 [*Halomonas zincidurans*] |

**Table S5** Sequences associated with Cas gene cluster found in the genome of A2

| **CAS CLUSTER** | |  |  |  |  |  |  |
| --- | --- | --- | --- | --- | --- | --- | --- |
| **#SequenceID** | **Cas-type/subtype** | **Gene status** | **System** | **Type** | **Begin** | **End** | **Strand** |
| Scaffold4_1_241 | *cas*1_TypeIF | mandatory | CAS-TypeIF | CDS | 254878 | 255852 | + |
| Scaffold4_1_242 | *cas*3-cas2_TypeIF | mandatory | CAS-TypeIF | CDS | 255849 | 259190 | + |
| Scaffold4_1_243 | *csy*1_TypeIF | mandatory | CAS-TypeIF | CDS | 259653 | 260999 | + |
| Scaffold4_1_244 | *csy*2_TypeIF | mandatory | CAS-TypeIF | CDS | 260996 | 261973 | + |
| Scaffold4_1_245 | *csy*3_TypeIF | mandatory | CAS-TypeIF | CDS | 261989 | 263023 | + |

**Table S6** Sequences associated with CRISPR regions found in the genome of A2

| **CRISPR_Id** | **CRISPR_Start** | **CRISPR_End** | **CRISPR_Length** | **Consensus_Repeat** | **Repeat_Length** | **Spacers_Nb** |
| --- | --- | --- | --- | --- | --- | --- |
| Scaffold1_1_1 | 17298 | 17428 | 130 | GACGACTGCTCGATTCTCGAGCTGAACGTGACCCTGCCCAAGGGCTGA | 48 | 1 |
| Scaffold1_1_2 | 145197 | 145334 | 137 | AGCGCATGCCGGGCCAGCCACTGGCCGAGGAGGACGAATCATGAGCCTGTCG | 52 | 1 |
| Scaffold1_1_3 | 238574 | 238697 | 123 | CCGGGGCATTACGATTCCGGCCGGCAGCCGTT | 32 | 1 |
| Scaffold1_1_4 | 378125 | 378222 | 97 | AGGCCGGCAAGTGGGTGGACCGCA | 24 | 1 |
| Scaffold2_1_1 | 12446 | 12576 | 130 | ACCAATAGCGACATCAGCGCCGCGTAGGTCAGGTTGTGGCGC | 42 | 1 |
| Scaffold2_1_2 | 63429 | 63514 | 85 | TGTGCCGGACGCGCTAGTTGTCC | 23 | 1 |
| Scaffold2_1_3 | 117313 | 117394 | 81 | ATCGGCGAGGCGATGTTCGAGCC | 23 | 1 |
| Scaffold2_1_4 | 187274 | 187407 | 133 | CCGAAGGGGTTGTAAGAGACTCGCTATGTGTTCGGCTT | 38 | 1 |
| Scaffold2_1_5 | 318843 | 318952 | 109 | CCTCGAAGGGCAAGCCGAGCAGCGCCC | 27 | 1 |
| Scaffold3_1_1 | 28309 | 28403 | 94 | GATGAGGCCGACCCCTGGGCCGA | 23 | 1 |
| Scaffold3_1_2 | 252104 | 252217 | 113 | CCTTCCCGGCGCCGCCGCTCCAGCCGCG | 28 | 1 |
| Scaffold3_1_3 | 369919 | 370032 | 113 | CTGGTGCCAGAGCCGCGCCAGACGCCCTATCCCG | 34 | 1 |
| Scaffold4_1_1 | 96979 | 97094 | 115 | GCCCCGGCGGCGGCGCCTACGGCGGCACC | 29 | 1 |
| Scaffold4_1_2 | 131827 | 131918 | 91 | GTGGATCAACCCGGCTCTCCATAACCCC | 28 | 1 |
| Scaffold4_1_3 | 263726 | 269693 | 5967 | GTTCGCTGCCGCCCAGGCAGCTCAGAAA | 28 | 99 |
| Scaffold4_1_4 | 307046 | 307145 | 99 | GGCGTTTAGGGCGTCGCATGGTGACG | 26 | 1 |
| Scaffold5_1_1 | 104625 | 104755 | 130 | CGCCTGATGGTGGCTCTCTATCCATCGGCGGGTATCT | 37 | 1 |
| Scaffold5_1_2 | 117844 | 117979 | 135 | GGCACCAGGCTGCGGTCACGGCTGATCCAGACGGCCAAGGCG | 42 | 1 |
| Scaffold5_1_3 | 196167 | 196270 | 103 | AGCCCATGATGCCGCGTCGCTCCT | 24 | 1 |
| Scaffold5_1_4 | 322497 | 322607 | 110 | CATGCGCAATTCTCGCCAGGCGCTGAAGAACCCCGAGC | 38 | 1 |
| Scaffold8_1_1 | 84306 | 84452 | 146 | GGGCGCGGGTCACTCATGGGCCAGTCTCTTGTTCGCAGTCATGGCAG | 47 | 1 |
| Scaffold8_1_2 | 182421 | 182535 | 114 | CGATTCTTTAGCCGCTGAAGCAGGCGCCTACCAT | 34 | 1 |
| Scaffold9_1_1 | 70399 | 70510 | 111 | TCGCCGGCCTCGTGGGCCAGTAGCCACTGCT | 31 | 1 |
| Scaffold10_1_1 | 100378 | 100466 | 88 | TCCCGCCGTCGGTCATTGTCCCG | 23 | 1 |
| Scaffold11_1_1 | 98253 | 98365 | 112 | TCCTGGCGCAGCGCCTCGAGCAGTGCATGG | 30 | 1 |

**Table S7** Biotechnological importance genes found in the genome of A2

| **Gene ID** | **Identity** | | ***Gene Name*** | **NR Description** | **KO ID** | **KO Description** |
| --- | --- | --- | --- | --- | --- | --- |
| **PHA synthesis** | |  |  |  |  |  |
| A2_GM000017 | | 81.5 | *phb*B | beta-ketoacyl-ACP reductase [*Halomonas anticariensis*] | K00023 | acetoacetyl-CoA reductase |
| A2_GM002200 | | 79 | *phb*C, *pha*C | class I poly(R)-hydroxyalkanoic acid synthase [*Halomonas anticariensis*] | K03821 | polyhydroxyalkanoate synthase |
| A2_GM000371 | | 84.7 | *pha*R | polyhydroxyalkanoate synthesis repressor PhaR [*Halomonas shengliensis*] | | |
| **Alpha-amylase** | |  |  |  |  |  |
| A2_GM002106 | | 46.7 | AMY, *amy*A, *mal*S | alpha-amylase [*Aquisalimonas asiatica*] | K01176 | alpha-amylase |
| **Arsenic resistance** | |  |  |  |  |  |
| A2_GM003132 | | 84.3 | *ars*H | arsenical resistance protein ArsH [*Halomonas* sp. A3H3] | K11811 | arsenical resistance protein ArsH |
| A2_GM003133 | | 91.6 | ACR3, *ars*B | arsenical-resistance protein [*Halomonas anticariensis*] | K03325 | arsenite transporter |
| A2_GM000558 | | 81.4 | *ars*A, ASNA1, GET3 | Arsenical pump-driving ATPase [*Halomonas chromatireducens*] | K01551 | arsenite/tail-anchored protein-transporting ATPase |
| A2_GM000749 | | 66.4 | ARSC1, *ars*C | arsenate reductase [*Halomonas taeanensis*] | K00537 | arsenate reductase |
| A2_GM001355 | | 64.7 | *pst*S | phosphate transport system substrate-binding protein [*Halomonas pantelleriensis*] | K02040 | phosphate transport system substrate-binding protein |
| A2_GM001356 | | 60.7 | *pst*C | hypothetical protein [*Halomonas anticariensis*] | K02037 | phosphate transport system permease protein |
| A2_GM001357 | | 72.2 | *pst*A | phosphate transport system permease protein [*Halomonas pantelleriensis*] | K02038 | phosphate transport system permease protein |
| A2_GM001358 | | 81.5 | *pst*B | phosphate ABC transporter ATP-binding protein [*Halomonas anticariensis* FP35 = DSM 16096] | K02036 | phosphate transport system ATP-binding protein |

**Table S8** Unique gene groups for adaptability to the environment of biotechnological relevance found in the genome of A2

| **Group** | **Gene** | **Description** | **ID** |
| --- | --- | --- | --- |
| **Sodium metabolism** | |  |  |
|  | *mrp*A_1 | Na(+)/H(+) antiporter subunit A | A2_00377 |
|  | *mrp*A_2 | Na(+)/H(+) antiporter subunit A | A2_00378 |
| **Molybdenum metabolism** | |  |  |
|  | *mod*A | Molybdate-binding protein ModA | A2_01236 |
|  | *mob*A | Molybdenum cofactor guanylyltransferase | A2_00631 |
|  | *mob*B | Molybdopterin-guanine dinucleotide biosynthesis adapter protein | A2_00632 |
|  | *moe*A_1 | Molybdopterin molybdenumtransferase | A2_00633 |
| **Flaggelar structure** | |  |  |
|  | *fli*D_1 | Flagellar hook-associated protein 2 | A2_00880 |
|  | *fli*D_2 | Flagellar hook-associated protein 2 | A2_00883 |
|  | *fli*F | Flagellar M-ring protein | A2_00890 |
|  | *fli*O | Flagellar protein FliO | A2_00899 |
|  | *flg*J | Peptidoglycan hydrolase FlgJ | A2_00905 |
|  | *flg*D | Basal-body rod modification protein FlgD | A2_00911 |
|  | *flg*A | Flagella basal body P-ring formation protein FlgA | A2_00914 |
|  | *flh*E | Flagellar protein FlhE |  |
| **Biofilm synthesis** | |  |  |
|  | *prs*E_1 | Type I secretion system membrane fusion protein PrsE | A2_01069 |
|  | *prs*D | Type I secretion system ATP-binding protein PrsD | A2_01070 |
| **Cytochrome *bo*3 complex** | |  |  |
|  | *cyo*A | Cytochrome bo(3) ubiquinol oxidase subunit 2 | A2_01452 |
|  | *cyo*B | Cytochrome bo(3) ubiquinol oxidase subunit 1 | A2_01453 |
|  | *cyo*C | Cytochrome bo(3) ubiquinol oxidase subunit 3 | A2_01454 |
|  | *cyo*D | Cytochrome bo(3) ubiquinol oxidase subunit 4 | A2_01455 |
|  | *cyo*E | Protoheme IX farnesyltransferase | A2_01456 |
|  | *cdh*R_5 | HTH-type transcriptional regulator CdhR | A2_01458 |
| **Tungsten metabolism** | |  |  |
|  | *tup*A | Tungstate-binding protein TupA | A2_01773 |
|  | *tup*C | Tungstate uptake system ATP-binding protein TupC | A2_01774 |
| **Acid resistance** | |  |  |
|  | *adi*C | Arginine/agmatine antiporter | A2_03467 |
|  | *adi*A | Biodegradative arginine decarboxylase | A2_03468 |
| **Iron metabolism** | |  |  |
|  | *fet*A | putative iron export ATP-binding protein FetA | A2_03175 |
|  | *fec*E_2 | Fe(3+) dicitrate transport ATP-binding protein FecE | A2_03145 |
|  | *nap*F | Ferredoxin-type protein NapF | A2_01932 |
|  | *nap*C | Cytochrome c-type protein NapC | A2_01936 |
| **Compatible solutes synthesis** | | |  |
|  | *pro*X | Glycine betaine/proline betaine-binding periplasmic protein | A2_02124 |
|  | *opu*AA | Glycine betaine transport ATP-binding protein OpuAA | A2_02126 |
|  | *nad*B | L-aspartate oxidase | A2_00560 |
| **Arsenic metabolism** | |  |  |
|  | *ars*A | Arsenical pump-driving ATPase | A2_00584 |
| **Copper metabolism** | |  |  |
|  | *cop*A_1 | Copper-exporting P-type ATPase | A2_00043 |
|  | *cop*A_2 | Copper-exporting P-type ATPase | A2_00336 |
| **Antibiotic resistance** | |  |  |
|  | *bep*G | Efflux pump membrane transporter BepG | A2_02500 |
|  | *bep*F | Efflux pump periplasmic linker BepF | A2_02501 |

**Figure S1** Cellular and colonial morphology of *Halomonas salifodinae* strain A2


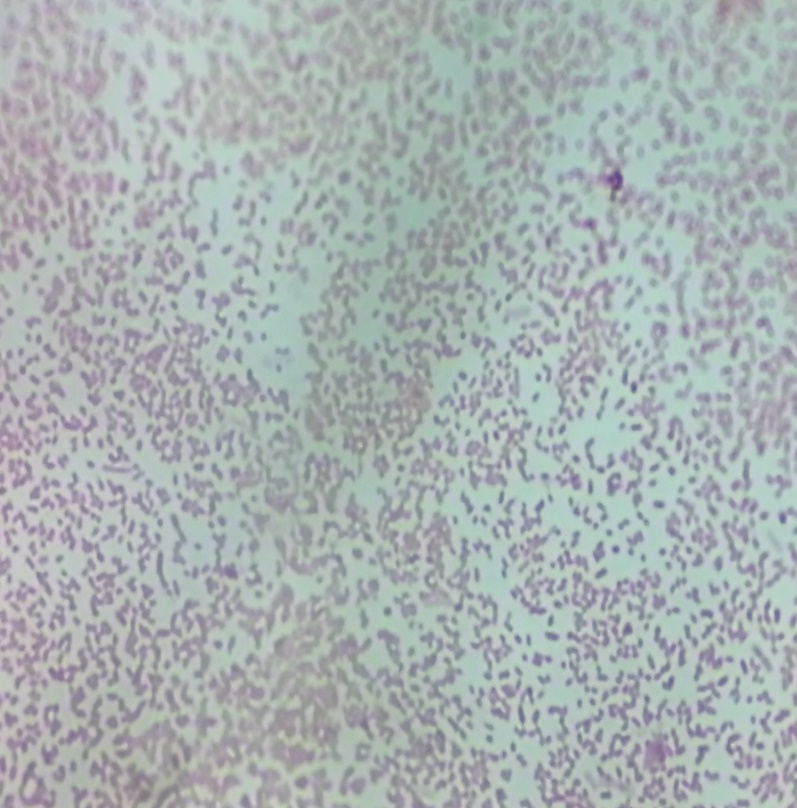

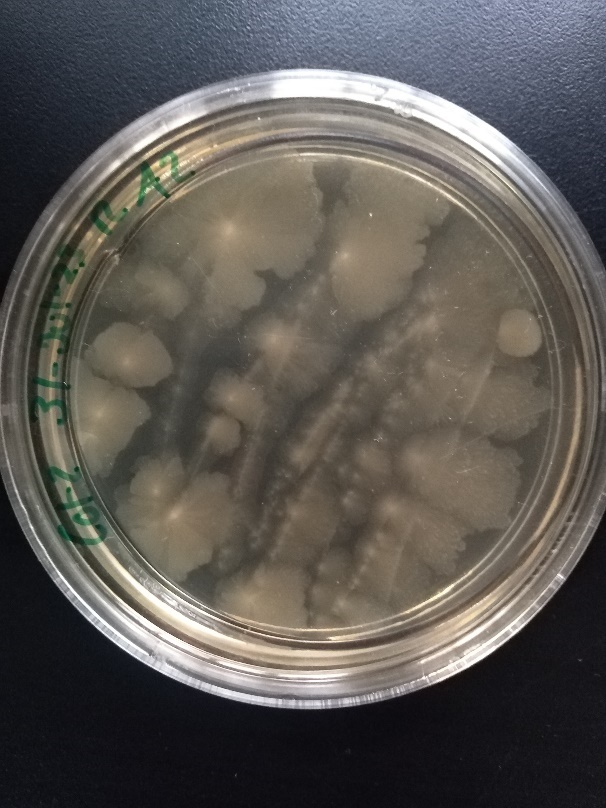


**
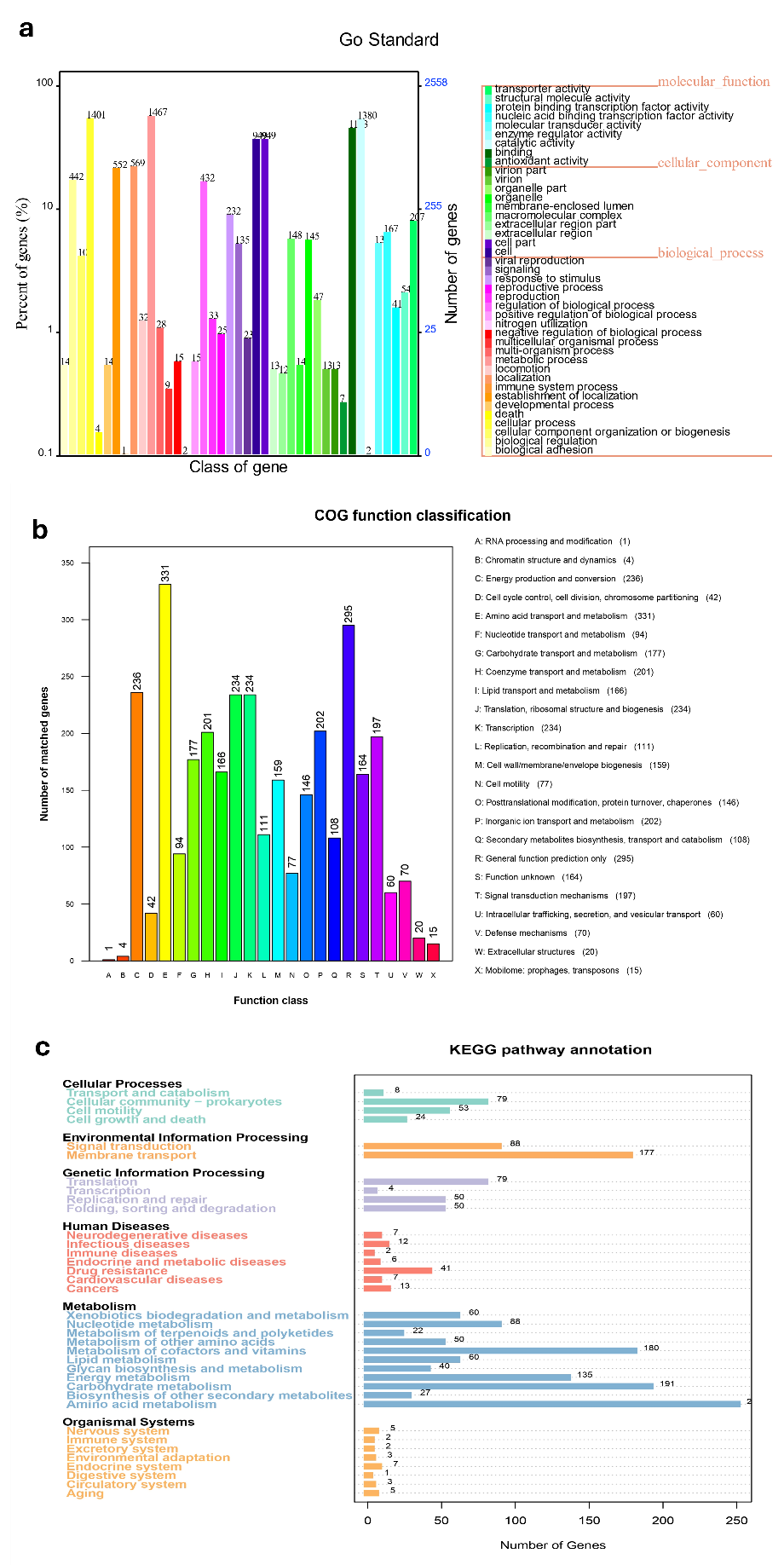
**

**Figure S2** Functional annotation of A2 in the different

databases used. **a** GO annotation; **b** COG annotation;

**c** KEGG annotation

**

**

**Fig. S3** Phylogenetic tree based on 16S rRNA sequences showing the phylogenetic position of A2 compared to *Halomonas* species from the TYGS database. Tree inferred with FastME 2.1.6.1 from GBDP distances calculated from 16S rRNA gene sequences. Branch lengths are scaled in terms of the GBDP d5 distance formula. The numbers above branches are GBDP pseudo-bootstrap support values > 60% from 100 replications, with an average branch support of 69.8%


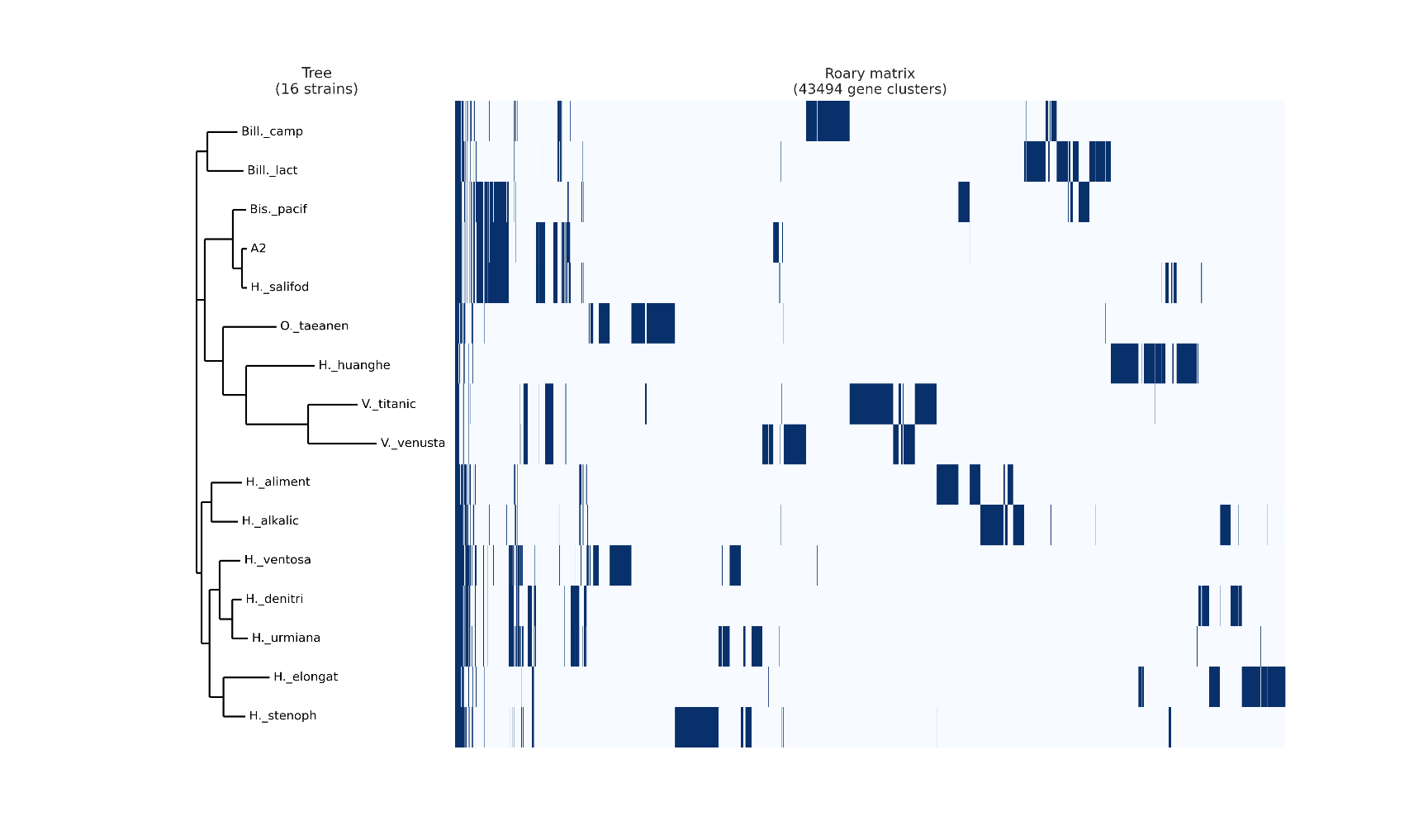


**Fig. S4** Pangenome matrix of the A2 genome against the closest species performed with the presence or absence of genes in Roary software

**Fig. S5** Organization of ectoine biosynthesis genes in A2 and other prokaryotes


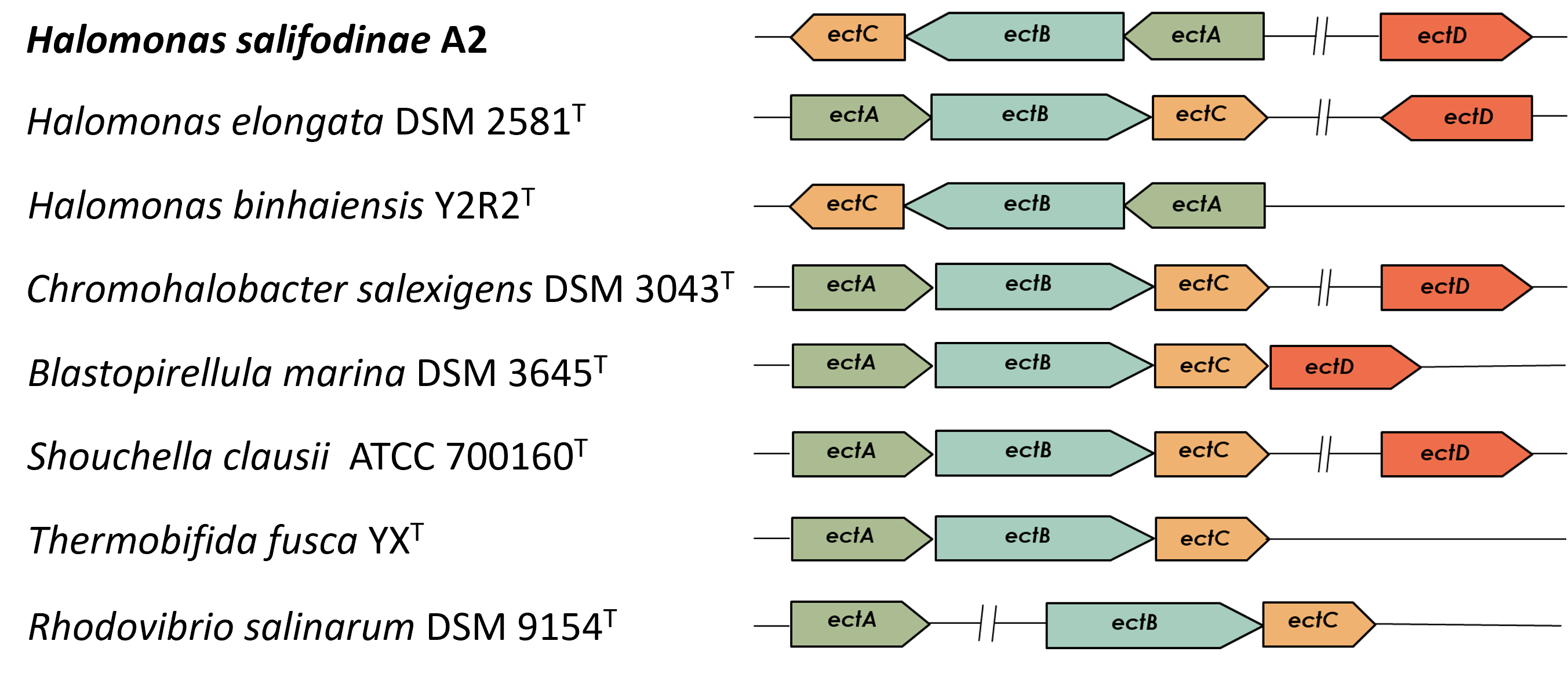

Supplement: Supplementary file 1 — Supplementary file1 (DOCX 4837 KB) [file 792_2025_1397_MOESM1_ESM.docx]
